# Design of Novel Enantiopure Dispirooxindolopyrrolidine-Piperidones as Promising Candidates toward COVID-19: Asymmetric Synthesis, Crystal Structure and *In Silico* Studies

**DOI:** 10.3390/molecules27123945

**Published:** 2022-06-20

**Authors:** Amani Toumi, Sarra Boudriga, Yasmine M. Mandour, Ahmed A. Mekki, Michael Knorr, Carsten Strohmann, Jan-Lukas Kirchhoff, Mansour Sobeh

**Affiliations:** 1 Laboratory of Heterocyclic Chemistry Natural Product and Reactivity (LR11ES39), Department of Chemistry, Faculty of Science of Monastir, University of Monastir, Monastir 5019, Tunisia; amanitoumi45@gmail.com; 2 School of Life and Medical Sciences, University of Hertfordshire Hosted by Global Academic Foundation, New Administrative Capital, Cairo 11578, Egypt; yasminemandour@yahoo.com (Y.M.M.); ahmed.abdelrasool@gaf.ac (A.A.M.); 3 Institut UTINAM-UMR CNRS 6213, Université Bourgogne Franche-Comté, 16 Route de Gray, 25030 Besançon, France; michael.knorr@univ-fcomte.fr; 4 Faculty of Chemistry and Chemical Biology, Inorganic Chemistry, Technische Universität Dortmund, Otto-Hahn-Strasse 6, 44227 Dortmund, Germany; carsten.strohmann@tu-dortmund.de (C.S.); jan-lukas.kirchhoff@tu-dortmund.de (J.-L.K.); 5 AgroBioSciences Research, Mohammed VI Polytechnic University, Lot 660-Hay MoulayRachid, Ben Guerir 43150, Morocco

**Keywords:** enantiopure spirooxindolopyrrolidine-piperidones, SARS-CoV-2, molecular docking, molecular dynamics, crystal structure, Hirshfeld analysis

## Abstract

Despite the effectiveness of COVID-19 vaccines, there is still an urgent need for discovering new anti-viral drugs to address the awful spread and transmission of the rapidly modifiable virus. In this study, the ability of a small library of enantiomerically pure spirooxindolopyrrolidine-grafted piperidones to inhibit the main protease of SARS-CoV-2 (M^pro^) is evaluated. These spiroheterocycles were synthesized by 1,3-dipolar cycloaddition of various stabilized azomethine ylides with chiral dipolarophiles derived from *N-*[*(S)*-(-)-methylbenzyl]-4-piperidone. The absolute configuration of contiguous carbons was confirmed by a single crystal X-ray diffraction analysis. The binding of these compounds to SARS-CoV-2 M^pro^ was investigated using molecular docking and molecular dynamics simulation. Three compounds **4a**, **4b** and **4e** exhibited stable binding modes interacting with the key subsites of the substrate-binding pocket of SARS-CoV-2 M^pro^. The synthesized compounds represent potential leads for the development of novel inhibitors of SARS-CoV-2 main protease protein for COVID-19 treatment.

## 1. Introduction

Since over two years, the COVID-19 pandemic resulting from the emergence of human coronavirus SARS-CoV-2, severe acute respiratory syndrome coronavirus 2, has caused devastating damage to the world. This significant global health threat disrupts people’s lives and has initiated an economic and social crisis [1,2]. As of March 2022, this highly transmissible respiratory disease caused over 433 million infections and resulted in 5.9 million fatalities worldwide [3]. Despite the development of numerous COVID-19 vaccines, there is still an urgent need for new complementary therapeutic agents for treatment and prevention of coronavirus infections to tackle the rapid transmission and resistance of the rapidly mutable SARS-CoV-2 [4,5]. Structural characterization of many SARS-CoV-2 drug targets allowed extensive use of molecular modelling and bioinformatics tools in investigating methods of diagnosis, prevention and treatment of SARS-CoV-2. Therefore, many computational studies have been conducted for repurposing and identifying novel promising therapeutic agents acting on various drug targets of SARS-CoV-2 [6,7,8]. 

**Figure 1:**
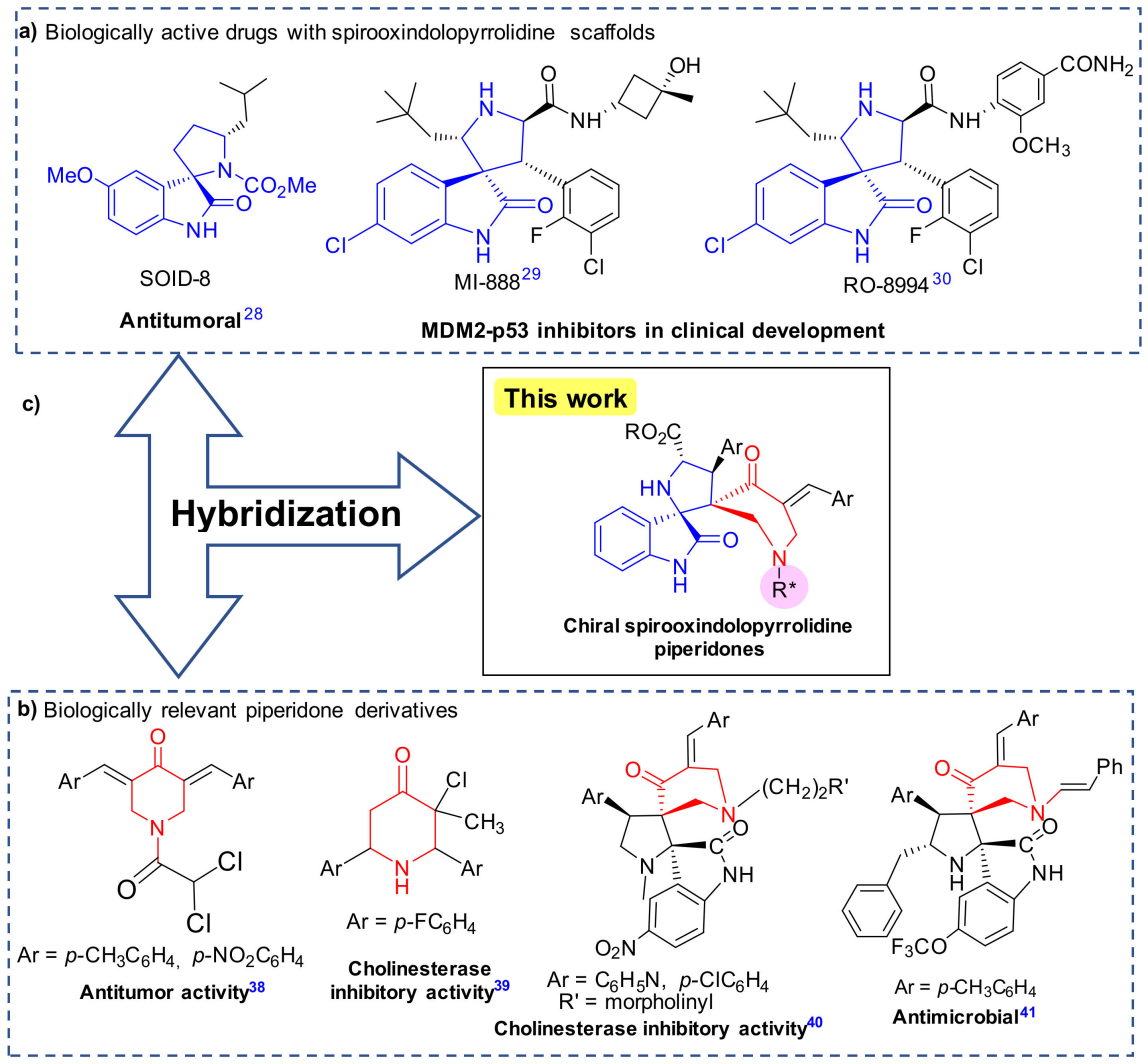
(**a**) Biologically relevant spirooxindolopyrrolidine derivatives; (**b**) Biologically active piperidone scaffolds; (**c**) Our target chiral spiroheterocycles combining the two motifs.

Nitrogen-based heterocycles represent a major key structural basis for numerous commercialized approved drugs [9]. The unique physicochemical properties of these heterocycles have garnered increased attention in designing and synthesizing new molecules, several of which display significant biological activities [10,11,12,13,14,15,16,17]. Recently, several libraries of *N-*heterocyclic compounds have been prepared and assessed for their anti-SARS-CoV-2 activities [18,19,20], resulting in a considerable number of drug candidates currently in clinical trials [21,22]. Oxindole and its derivatives are among the *N*-heterocycles with intriguing biological and therapeutic values [23]. Among their various pharmaceutical applications, they have been described as tyrosine kinase inhibitors [24], anticancer and antiangiogenic agents [25], cardiotonic and inhibitors of type IV Cyclic AMP Phosphodiesterase [26]. Recently, spirooxindolopyrrolidine, a spirancic subclass of this family, have aroused great interest for designing innovative drug-like compounds [27]. Considerable efforts have been undertaken to employ this spirocyclic motif as a core structure in synthesizing novel drug candidates, several of them exhibiting promising biological and pharmacological profiles. For instance, the spirocyclic pyrrolidine SOID-8 has been reported to inhibit growth and induce apoptosis in human melanoma cells. (Figure 1) [28]. 

**Scheme 1:**
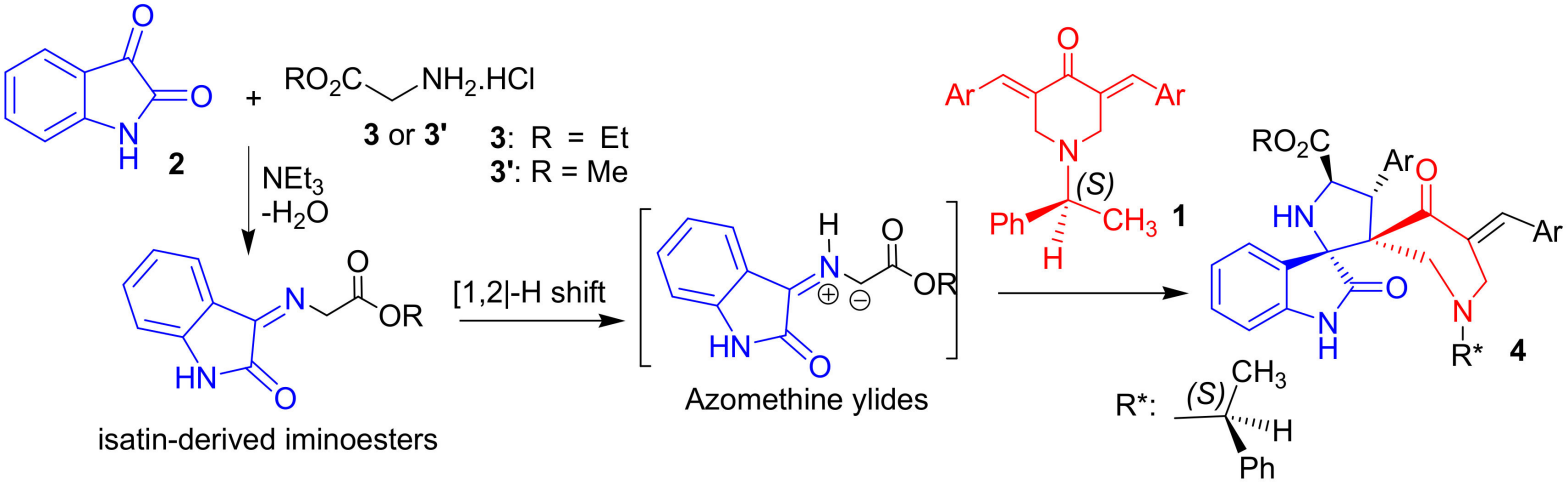
Synthesis scheme for the targeted chiral spiroheterocycles **4**.

MI-888 and RO-8994 (Figure 1) have been described as potent inhibitors of MDM2–p53 interaction and are undergoing clinical assessment for cancer therapy [29,30] (see also https://en.wikipedia.org/wiki/Mdm2, accessed on 20 May 2020). In addition, several derivatives have been reported as anti-amyloidogenic [31], antimycobacterial and antimicrobial [32,33], antiviral [34], anti-acetylcholinesterase [35], and anti-inflammatory agents [36]. The inherent three-dimensional nature of these spiroheterocycles and the good balance between its flexibility and conformational rigidity enable optimal binding interactions with target proteins, thus, increasing the chances of discovering novel drugs [37]. Similarly, piperidone and their *α,β*-unsaturated ketones were previously reported to display a plethora of biological properties [38,39] (Figure 1). Recent investigations revealed spirooxindolopyrrolidine cores embedded with 4-piperidone rings as a new class of bioactive polyheterocyclic spiro-compounds with promising anti-cholinesterase [40], antifungal [41], and anti-inflammatory activities [36], in addition to their remarkable anticancer properties [42] (Figure 1). Based on these findings, the racemic forms of the aforementioned spiroheterocycles were constructed by multicomponent [3+2] cycloaddition of achiral arylidene-piperidones as dipolarophiles with isatin-derived azomethine ylides as dipoles. Considering the rigid structure of the aforementioned scaffolds, obtaining them in enantiopure form appears quite difficult. To the best of our knowledge, no approaches for synthesizing chiral spirooxindolopyrrolidines have been reported so far. Using our previous experiences on the design spiroheterocycles [43,44,45,46], we describe herein, for the first time, the straightforward asymmetric synthesis of a novel library of chiral spiroheterocycles comprising the two pharmacophores spirooxindolopyrrolidine and piperidone within a unique tetracyclic scaffold (Figure 1). The strategy relies on a [3+2] cycloaddition of enantiopure *(E,E)-*3,5-bisarylidene-*N*-[(*S*)-(-)-methylbenzyl]-4-piperidones **1a**–**e** with azomethine ylides, generated *via* thermal [1,2]-prototropy of the isatin-derived iminoesters. The latter were formed from condensation of isatin **2** and glycine aminoester **3** or **3′** (Scheme 1). Furthermore, the binding of these chiral spiroheterocycles to the main protease of SARS-CoV-2 (M^pro^) was explored by *in silico* molecular docking and molecular dynamics simulations followed by RMSD, free energy calculation and H-bond analysis to evaluate the potential of these compounds in medicinal chemistry 

## 2. Results and Discussion 

### 2.1. Synthetic Chemistry

**Scheme 2:**
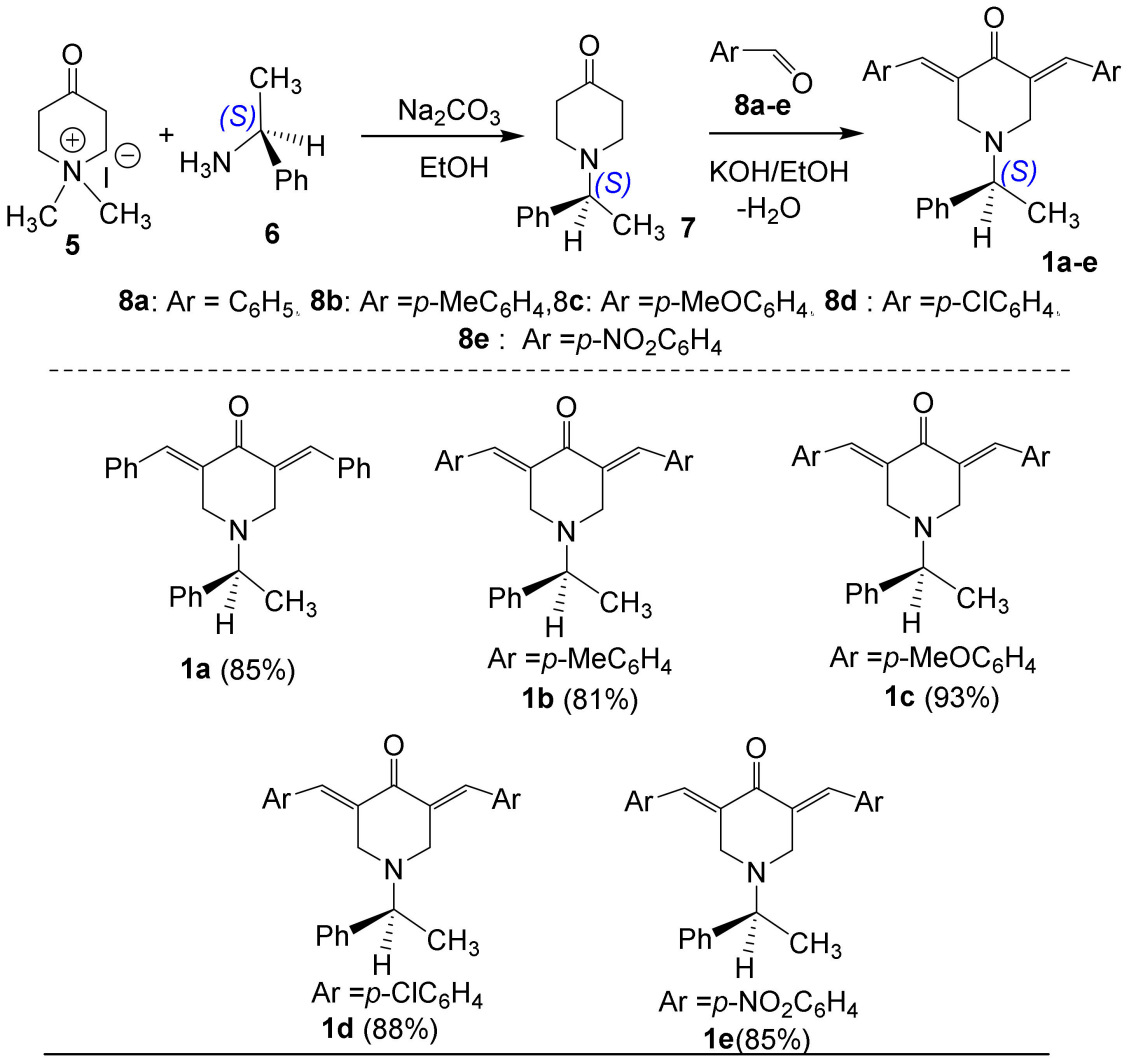
Synthetic route to *(S)-*(-)*-(*1-methylbenzyl)-3,5*-(E,E)-*bisarylidene-4-piperidones **1a**–**e**.

At the onset, the starting material *N*-[(*S*)-(-)-methylbenzyl]-4-piperidone 7 was synthesized by the *transamination* reaction of *N*-methyl-4-piperidone methiodide salt **5** with commercially available (*S*)-(−)-1-phenylethylamine **6** (Scheme 2), according to a previously published procedure [47]. Then, a series of enantiopure *(E,E)-*3,5-bisarylidene-*N*-[(*S*)-(-)-methylbenzyl]-4-piperidones **1a**–**e** was prepared with excellent yields (81–93%) via Knoevenagel condensation reaction between *N*-[(*S*)-(-)-methylbenzyl]-4-piperidone **7** and various aromatic aldehydes **8** bearing both electron-donating or withdrawing substituents at the *para*-position. The reaction was performed in alkaline medium at room temperature. 

**Figure 2:**
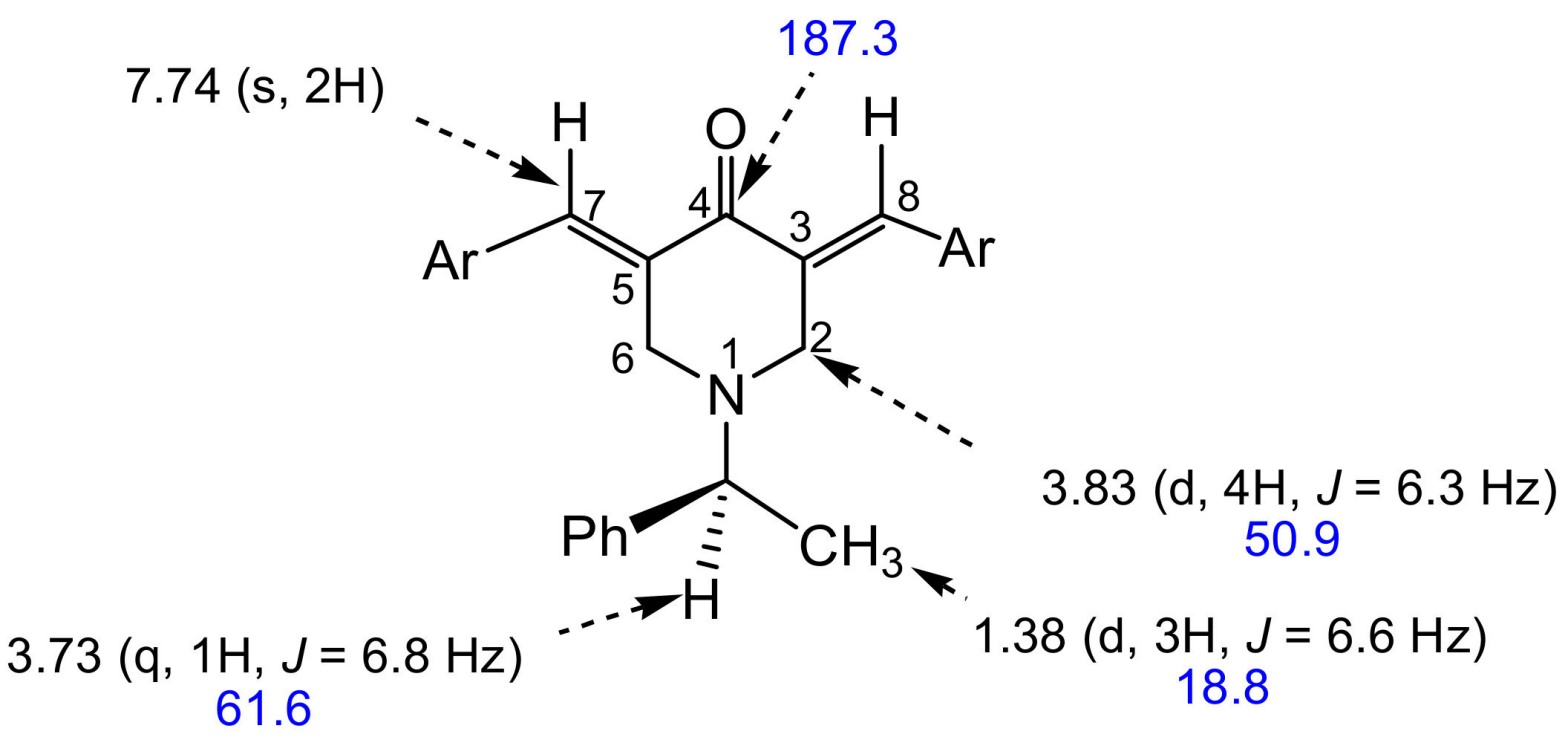
Selected ^1^H (black) and ^13^C (blue) chemical shifts of optically active compound **1d**.

The proposed structure of the five exocyclic alkenes obtained as yellow solids is in accordance with their 1D and 2D NMR spectroscopic data. Relevant ^1^H and ^13^C chemical shifts of **1d** are given below in Figure 2.

The *(E,E)* configuration of the two exocyclic carbon-carbon double bonds in exocyclic alkenes **1a**–**f** was confirmed using a NOESY experiment (Figure S10, in the Supplementary Materials). The 2D NOESY spectrum of **1d** shows NOE contacts between aromatic protons and the H-2 and H-6 resonating at δ 3.83 ppm as a doublet, indicating that they are close to each other. However, no NOE correlations were detected between the olefinic protons H-7 and H-8 and methylene protons of the piperidone ring. 

**Scheme 3:**
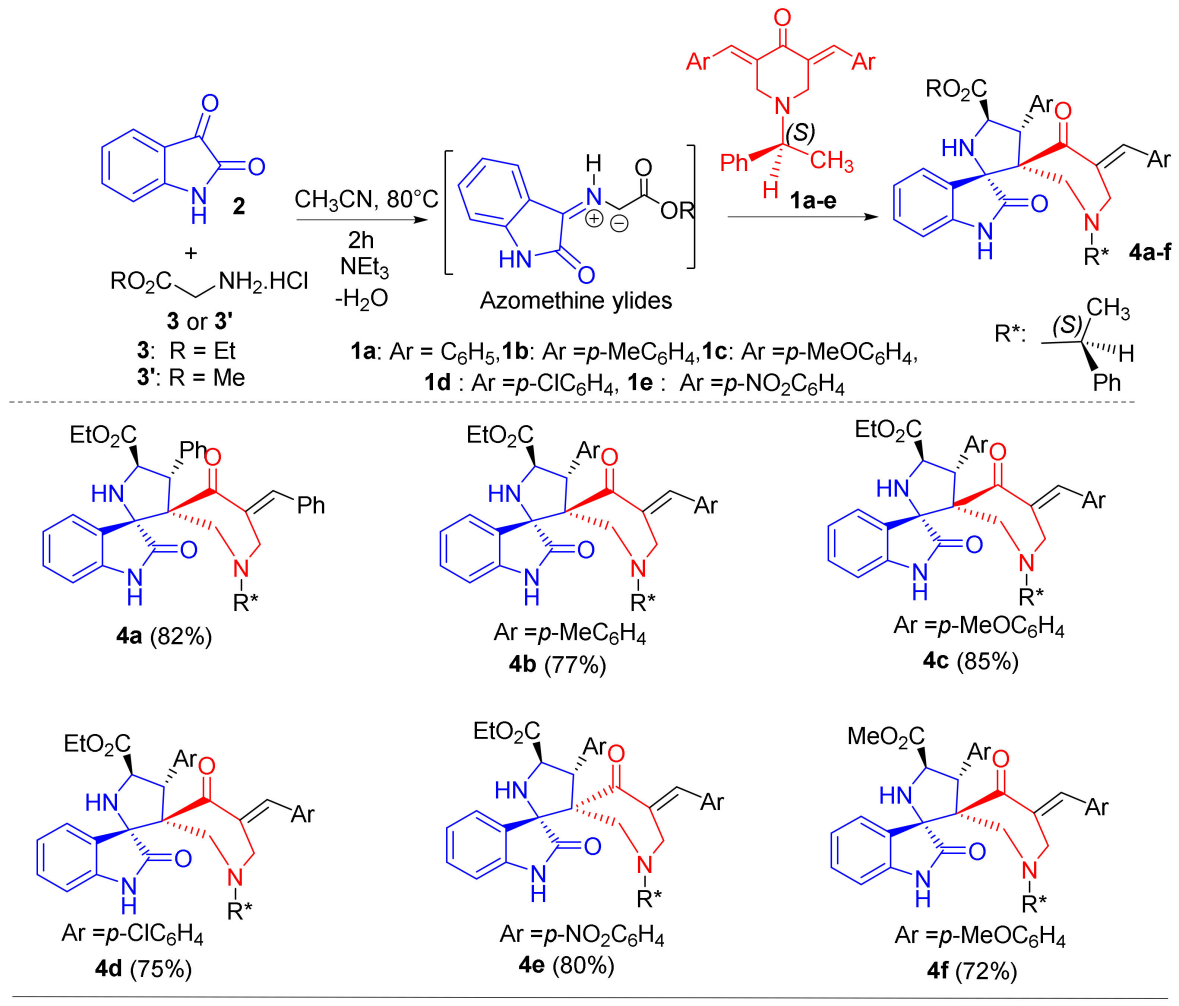
Synthesis of enantiopure spirooxindolopyrrolidine-piperidones **4**.

After spectroscopic confirmation of the chiral nature and optical activity of compounds **1**, the have been employed in a second step as dipolarophiles for a cyloaddition sequence under chiral conditions. According to our previously published procedure for the efficient synthesis of spiro[2,3’]-oxindole-spiro[3,3”]thiochroman-4”-one-4-aryl-5-carboxyethoxypyrrolidines, an equivalent amount of optically active *(E,E)-*3,5-bisbenzylidene-*N*-[(*S*)-(-)-methylbenzyl]-4-piperidone **1a**, isatin **2** and chlorhydrate of glycine ethyl ester **3** were reacted in acetonitrile at 80 °C for 2 h in the presence of triethylamine. The multicomponent reaction (MCR) proceeded smoothly to afford the desired chiral spirooxindolopyrrolidine **4a** (Scheme 3). Moreover, TLC monitoring and 1D NMR analysis of the reaction crude clearly indicated the formation of such compounds as the sole diastereoisomer (Scheme 3). Next, the scope of the MCR was extended with various enantiomerically pure dipolarophiles **1** bearing a NO_2_ group or electronically different substituents (4-Me, 4-OMe and 4-Cl) on the phenyl ring. As shown in Scheme 3, this process affords new chiral spirooxindolopyrrolidines-grafted piperidones **4b**–**e** with good yield (75–85%) along with high diastereoselectivity (Scheme 3). Notably, the MCR also worked efficiently when using glycine methyl ester **3′** as an alternative glycine aminoester, instead of **3**, affording the corresponding optically active spiroheterocycle **4f** in 72% yield (Scheme 3). Note that we failed to activate the remaining C=C–Ar bond of **4** for a further cycloaddition to extend to a pentacyclic framework. 

#### 2.1.1. Spectroscopic and Crystallographic Characterization of Cycloadducts 4

**Figure 3:**
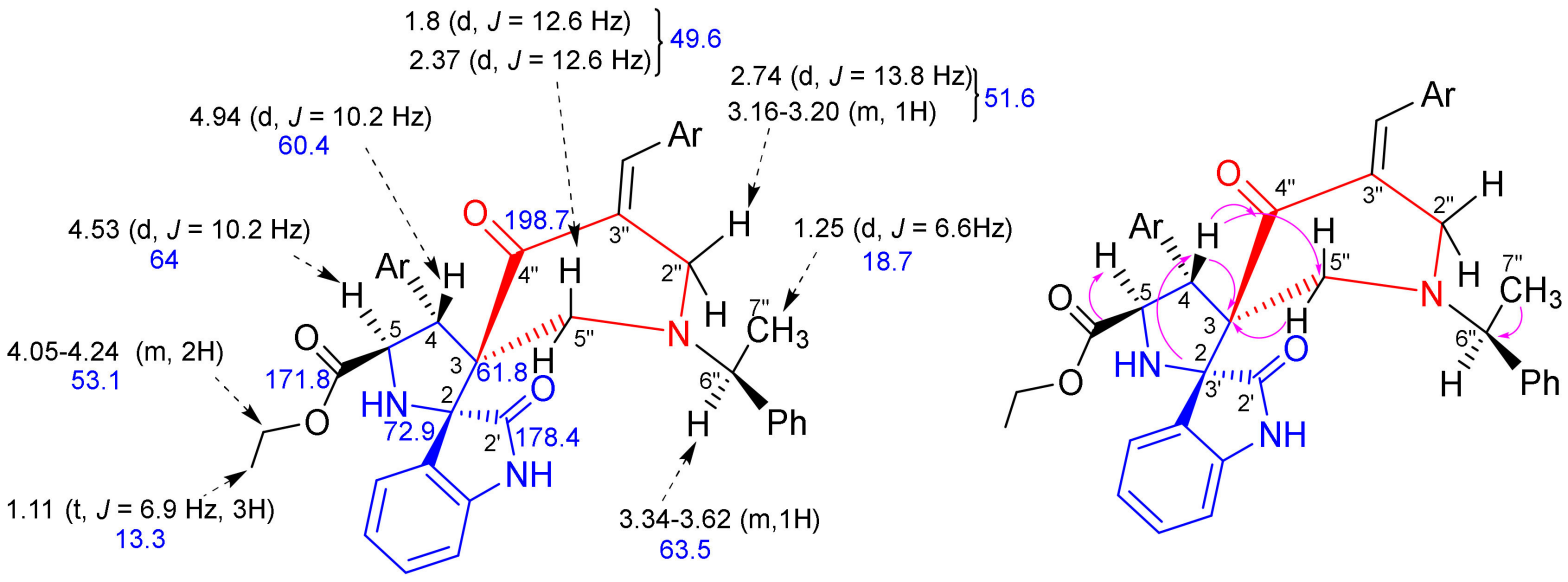
^1^H (black) and ^13C^ NMR (blue) chemical shifts along with selected HMBC correlations of **4c**.

The structure of the spiropyrrolidines **4** was determined by 1D and 2D NMR spectroscopic techniques (HMQC and HMBC), as discussed for a representative example **4c** (Figure 3). 

In the ^1^H NMR spectrum of **4c** (Figure S17 in the Supplementary Materials), the observation of two characteristic doublets at δ 4.53 and 4.94 ppm (*J* = 10.2 Hz) assigned to the mutually coupled hydrogens H-5 and H-4 of the pyrrolidine ring clearly confirms the formation of a new pyrrolidine ring. The two signals were distinguished from the Heteronuclear Multiple-Bond Correlations (HMBC) (Figure S21 in the Supplementary Materials) of proton H-4 with (*i)* the spiro carbons C-2 and C-3 at 72.9 and 61.8, respectively, and *(ii)* carbonyl carbon C-4” at 198.7 ppm (Figure 3). From the HMQC spectrum (Figure S20 in the Supplementary Materials), the carbon signals at 64.0 and 60.4 ppm can be readily assigned to C-5 and C-4, respectively. Furthermore, the multiplicity of H-4 and H-5 protons is particularly relevant to ascertain the regiochemistry of the 1,3-dipolar cycloaddition reaction (Scheme 1). In addition, the H-4 and H-5 protons are in *trans* relationship based on the value of the *^3^J* coupling constant (*J* = 10.2 Hz) and on our previous reports on related spiropyrrolidines [44]. Further, H-4 shows a correlation with a methylene carbon at 49.6 ppm assigned to the C-5”. The HMQC spectrum assigns the diastereotopic 5”-CH_2_ hydrogens to the two doublets at 1.8 and 2.37 ppm (*J* = 12.6 Hz), which show HMBCs with the carbons C-3 and C-4” at 61.8 and 198.7 ppm, respectively.

Further, one of the 2”-CH_2_ hydrogens of the piperidone ring appears as doublet at 2.74 ppm with *J* = 13.8 Hz and the other one as a multiplet between 3.16 and 3.2 ppm, whereas the HMQC correlation assigns the carbon signal at 51.6 ppm to C-2”. The doublet at δ 1.25 ppm (*J* = 6.6 Hz) can be assigned to the methyl protons 7”-CH_3_. The multiplet between δ 6.69 and 8.15 ppm is attributed to the aromatic and benzylic proton. In addition, the occurrence of three low-field peaks at δ 171.8, 178.4 and 198.7 ppm indicates the presence of three carbonyl groups belonging to the dipole and piperidone fragments. The methoxy, methylene, methine, and spiro carbons were assigned by DEPT-135 spectrum (Figure S19 in the Supplementary Materials). 

**Figure 4:**
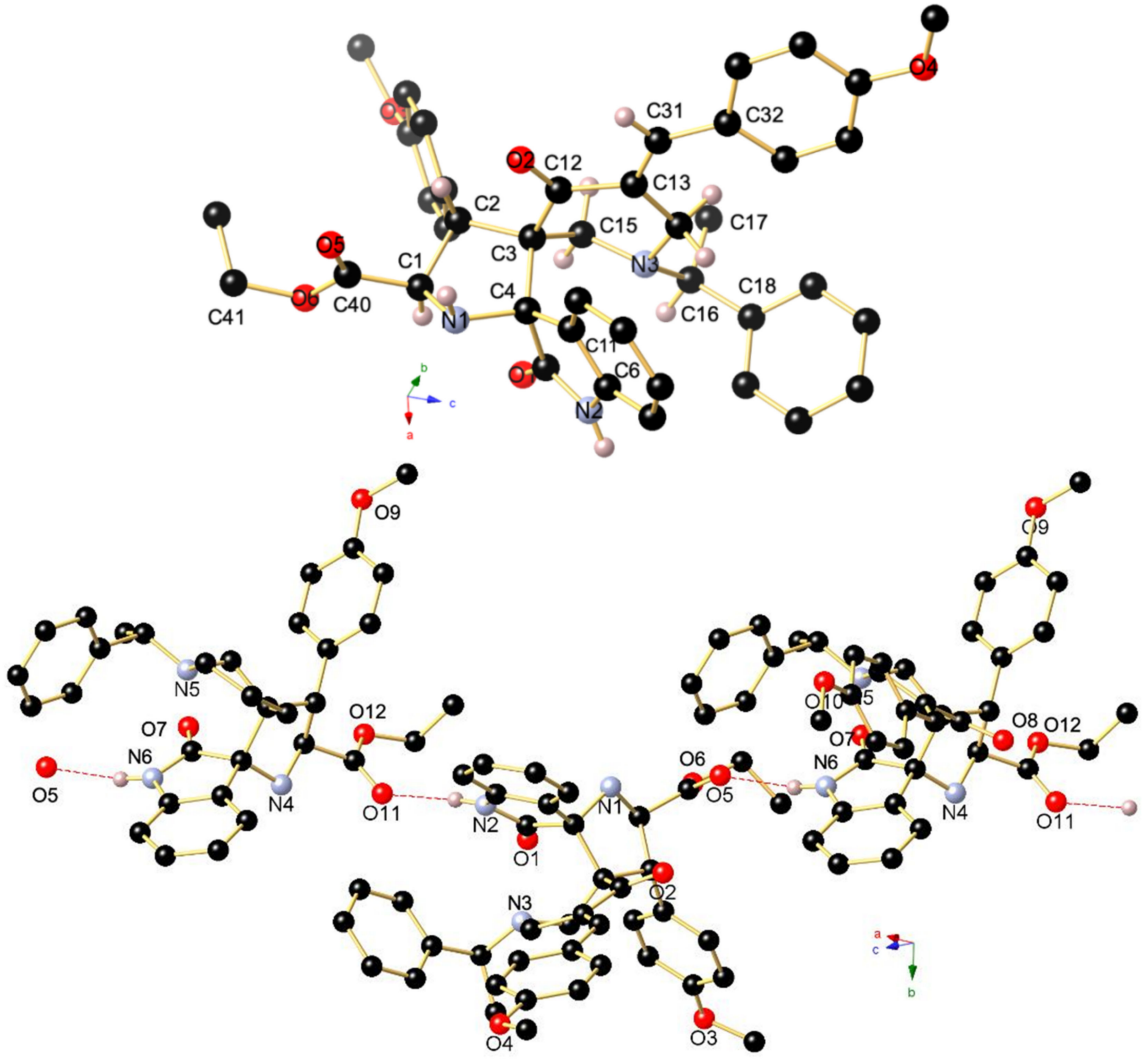
(Top) Ball and Sticks presentation of compound **4c** in the crystal. Only stereochemically significant hydrogen atoms are presented. Only one of the two independent molecules in the unit cell is shown. Selected bond lengths (Å) and angles (°): C1–N1 1.470(2), N1–C4 1.478(2), C1–C2 1.553(2), C2–C3 1.563(2), C3–C4 1.601(2), C4–C5 1.564(2), C5–O1 1.221(2), C5–N2 1.359(2), N2–C6 1.393(3), C6–C11 1.404(3), C11–C4 1.519(2), C3–C12 1.532(2), C12–O2 1.226(2), C12–C13 1.494(2), C14–N3 1.462(2), N3–C15 1.456(2), C13–C31 1.355(3); C1–N1–C4 103.87(14), N1–C1–C2 105.65(14), C1–C2–C3 103.41(13), C3–C12–C13 116.31(15), C13–C14–N3 115.37(14), C14–N3–C15 113.38(14), N3–C15–C3 107.79(14), C15–C3–C12 106.88(14), C12–C13–C31 116.85(16), C13–C31–C32 129.99(17). (Bottom) Association of the two independent compounds in the packing through intermolecular N–H···O hydrogen bonding.

The absolute configuration of the optically active spirooxindolopyrrolidine stereocenters was unequivocally corroborated after determination of the single-crystal X-ray structure of spiroheterocycle **4c**, whose molecular structure is depicted in Figure 4. The X-ray analysis data reveals that the absolute configuration of **4c** is 2*R*, 3*R*, 4*S*, and 5*S*. In addition, from Figure 4, the chiral auxiliary and the exocyclic double bond of 4-piperidone adopt a half-chair conformation and are oriented pseudo equatorially. This diastereofacial selectivity is explained by the *exo* approach of the *(Z,E)-*dipole, on the less hindered upper face of the dipolarophile. 

The asymmetric unit of **4c** contains two independent molecules (with slightly different bond lengths and angles), which are mutually associated through a strong intermolecular N–H ····O=C hydrogen bonding occurring between N2–H2 and O11^1^ with *d*(N2-H2····O11^1^ 1.96(3) Å, the N2–H2····O11 angle being 169(3)°. Furthermore, a second intermolecular hydrogen bond is formed between N5–H5 and O5 (*d*(N5–H5····O5 1.98(3) Å), thus generating a supramolecular 1D ribbon as shown in Figure 4 (Bottom).

#### 2.1.2. Hirshfeld Surface Analysis 

**Figure 5:**
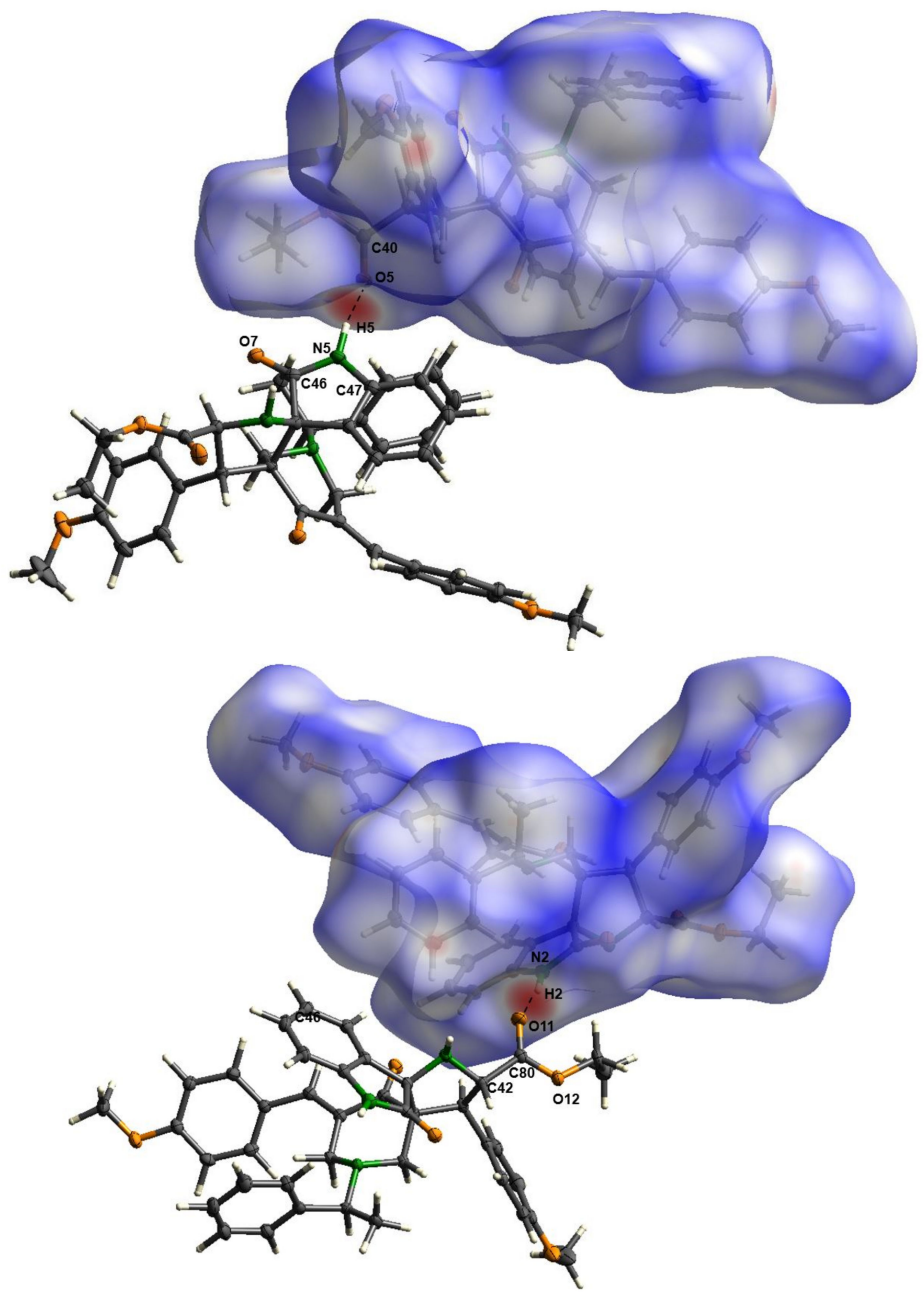
Hirshfeld surface analysis of molecule **4c** highlighting close contacts in the crystal structure between the two independent compounds. Selected close contacts [Å] and angles [°]: N1–H1 0.91(3), N2–H2 0.90(3), N4–H4 0.93(3), N5–H5 0.87(3), O1–C5 1.221(2), O7–C46 1.221(2), C3–C4 1.600(2), C45–C46 1.558(2); N5–H5···O5 169(3), N2–H2···O11 169(3), C30–H30b···O8 141(2), C63–H63···O9 164(2). Symmetry codes: (i) x, y, z; (ii) –x, 1/2–y, –z.

To better clarify the presence of weak intermolecular contacts occurring in the crystalline state, that may be complementary and relevant to the docking simulation shown in Section 2.2, a Hirshfeld surface analysis [48] was carried out utilizing the *CrystalExplorer21* software package [49]. For molecule **4c**, the Hirshfeld surface is mapped over *d*_norm_ in the range from –0.5804 to 1.5493 (arbitrary units). As presented in Figure 5, the characteristic red spots highlight the close intermolecular contacts. Significant N–H**···**O and weaker C–H**···**O interactions can be seen, what can be inferred from the individual fingerprint spectrum. The strongest interactions are N–H···O contacts, such **as** N2–H2···O11 and N5–H5···O5, which can be classified as very strong due to the short length below 3 Å and angles of about 170°. In addition, compound **4c** has a suitable supramolecular propensity to interact with other polar molecules. The C–H···O contacts, such as C30–H30b···O8 and C63–H63···O9, are much weaker and form only hydrogen bonds of moderate strength.

### 2.2. Molecular Dynamics and Binding Modes Analysis

**Figure 6:**
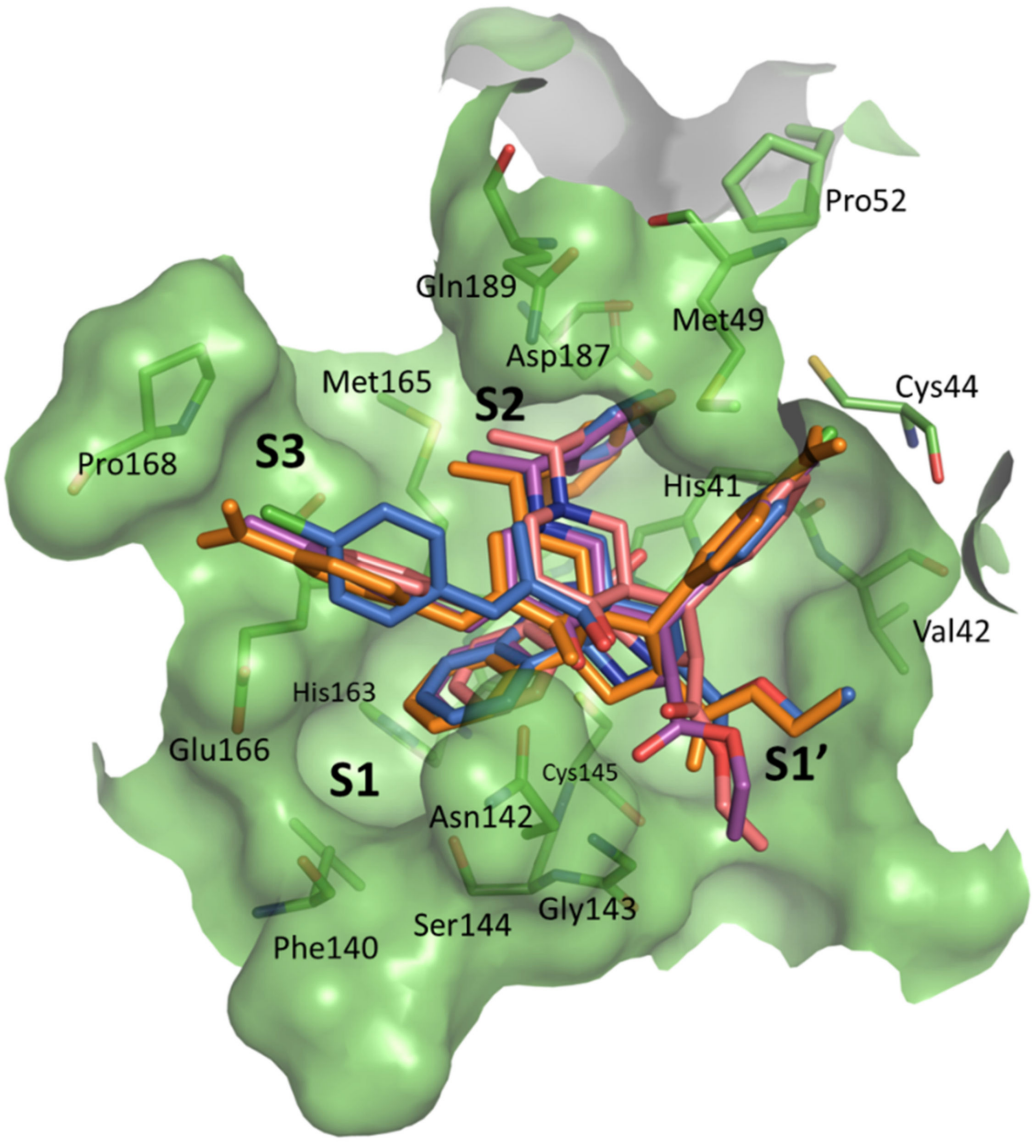
Graphical representation of the substrate-binding pocket of SARS-CoV-2 M^pro^ [50] revealing S1′–S3 subsites. Overlay of the docked poses of compounds **4a** (coral), **4b** (magenta), **4d** (blue) and **4e** (orange).

The binding mode of the six compounds was elucidated by molecular docking simulations in the active site of SARS-CoV-2 M^pro^ (PDB: 6W63) [50] using GOLD 5.8 [51,52] (Cambridge Crystallographic Data Centre, Cambridge, UK), as reported previously [53]. Interestingly, the docking results of four compounds **4a**, **4b**, **4d** and **4e** converged to a similar binding mode. The oxindole ring occupies the S1 subsite while the pyrrolidine ring with its 4-substituted aryl group occupies the S1′ subsite mimicking the imidazole ring of the cocrystallized ligand. The *N*-[(S)-(-)-methylbenzyl]-group appears, extending to the deep S2 subsite. Finally, the substituted 3-arylidene of the piperidone ring resides in the S3 subsite. The limited size of the S1′ subsite will not be able to accommodate the extended methoxy phenyl group of the pyrrolidine ring found in the structures of **4c** and **4f** which explains the failure of these latter compounds to exhibit the same binding mode (Figure 6). 

**Figure 7:**
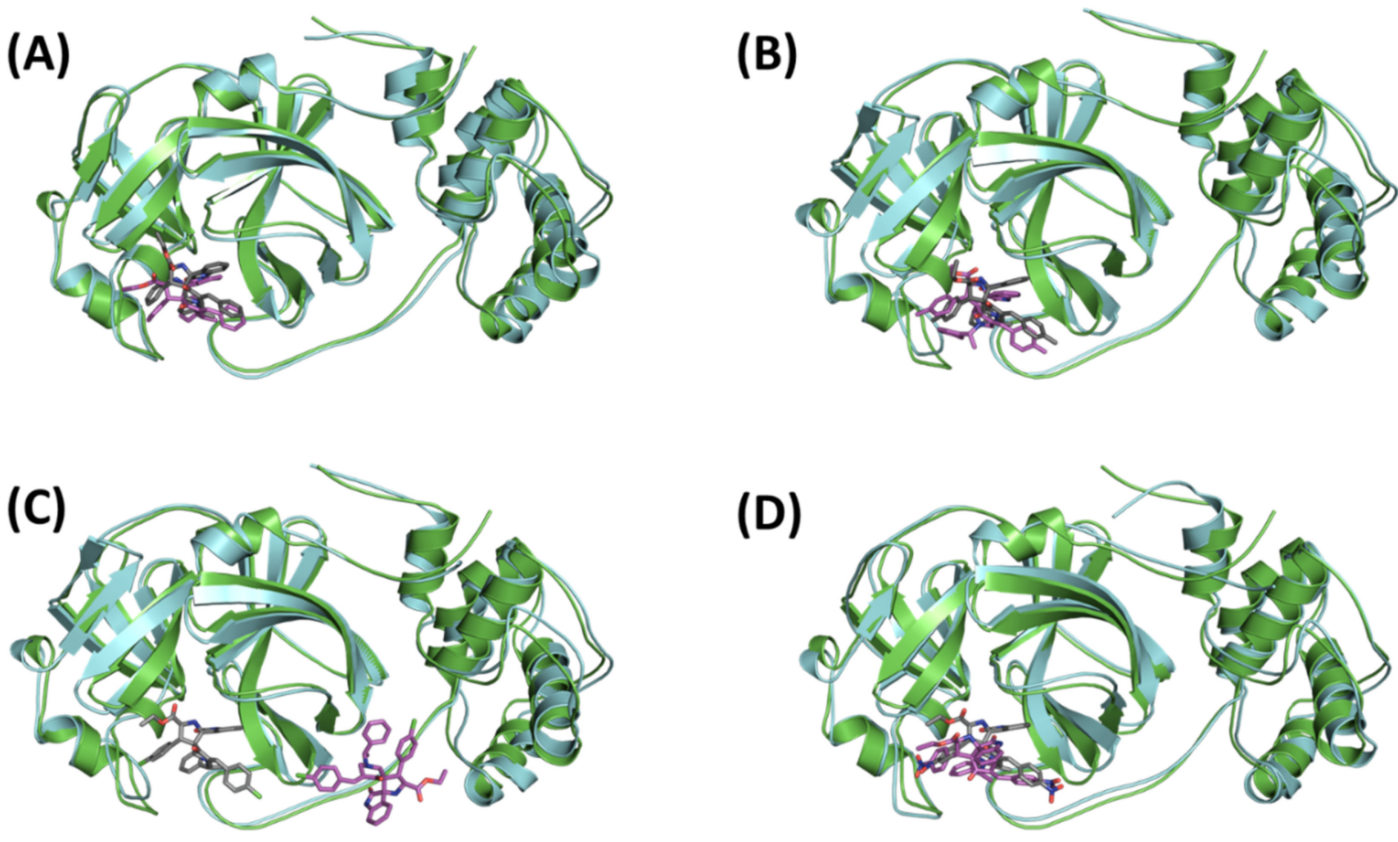
Overlay of the crystal structure (light cyano) and a representative MD structure (green cartoon) of SARS-CoV-M^pro^ bound to the docked pose of (**A**) compound **4a** (**B**) compound **4b** (**C**) compound **4d** and (**D**) compound **4e**. For each ligand, the initial docked ligand is presented as grey sticks, while that of the representative MD structure is presented as magenta sticks.

To further confirm the stability of these proposed binding modes, 80 ns molecular dynamics simulations were conducted for each M^pro^-ligand complex using the AMBER software package [54]. In total, four ligands were studied (**4a**, **4b**, **4d** and **4e**) to check the stability of their binding modes. Visual examination of the resultant trajectories showed the three compounds **4a**, **4b** and **4e** capable of retaining GOLD docked pose (Figure 7). On the other hand, visual inspection of the resultant trajectory of compound **4d** showed it completely detached from the binding site at the beginning of the production run and failing to reach a stable binding mode within the conducted simulation. Accordingly, compound **4d** was not further considered in this study (Figure 7C). It is worth mentioning that the dimeric state of M^pro^ is the functional form of this protease protein [55]. The absence of the second chain of M^pro^ could attribute to the failure of compound **4d** to exhibit a stable binding mode during the simulations. Future simulation of compound **4d** in the dimeric state of M^pro^ could confirm whether its egress was due to the absence of this second chain or not. 

**Figure 8:**
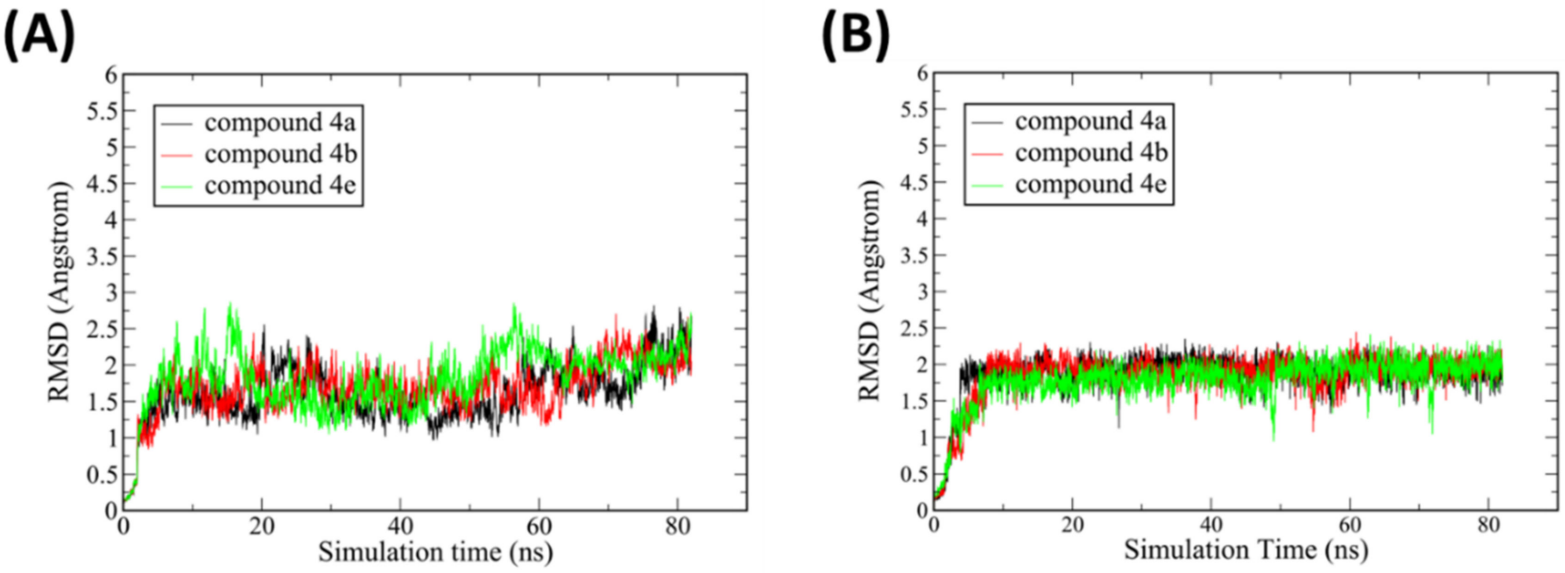
Time evolution of RMSD of the entire trajectory consisting of structures sampled in intervals of 20 ps. The plot depicts RMSD values based on (**A**) protein backbone atoms and (**B**) ligand heavy between the trajectory frames and the starting geometry.

For compounds **4a**, **4b** and **4e**, examining the time evolution of the root mean square deviation (RMSD) values of the protein’s backbone atoms showed stability of the protein architecture evident by its minor RMSD fluctuations which levelled off at around 2.5 Å after almost 25 ns. In addition, the ligands’ binding modes appeared to exhibit high stability where the RMSD levelled off at around 2 Å after almost 25 ns, confirming the binding modes proposed by GOLD docking experiments (Figure 8). All the collected MD samples were used to generate an average representative of the respective complex, and the sample showing the highest structural similarity with this average structure was selected to best represent the conformational space of the complexes. As shown in Figure 7, the average MD structures showed an excellent match to their parent X-ray structures.

H-bond interactions in each complex were detected and their abundance determined throughout the production trajectory with the results provided in Table 1. It is apparent that all three compounds exhibit H-bond interactions with residues at the protease S1 and S2 subsites. For the S1 subsite, two residues appeared responsible for maintaining interactions with the docked ligands, Asn142 and Gly143. The three compounds showed H-bond interactions with the side chain of Asn142 while Gly143 showed interactions with compounds **4a** and **4b** only.

In addition, all three ligands display interactions with the backbone of His164 in the S2 subsite. Interestingly, compound **4b** showed an additional interaction with the catalytic His41 residue in the S2 subsite. Overall, compound **4b** showed superiority in maintaining its interactions compared to the other two compounds, where its H-bonds with His41 and His164 in the S2 subsite were persistent in 71% and 62% of the production MD trajectory snapshots, respectively, while its interactions with Gly143 and Asn142 in S1 subsite appeared in 43% and 21% of the production MD trajectory snapshots, respectively.

To compare the binding affinities of these compounds, we used the MM-GBSA method [56] to calculate the binding free energy of the ligand–M^pro^ complexes as they evolved during their 80 ns simulation. For each complex, snapshots were sampled at a regular interval of 10 ps from the last 10 ns trajectory, resulting in 1000 frames for the MM-GBSA calculations. The binding free energies of the complexes are provided in Table 2 and compared to the previously reported binding affinity of the cocrystallized ligand X77 [53]. Compounds **4a**, and **4e** displayed relatively similar binding affinity scores of −30.7, and −30.4 kcal/mol, respectively, while compound **4b** showed a higher binding affinity of −35.2 kcal/mol. The relative binding affinities of the compounds confirmed the superiority of compound **4b** in accordance with the results of the H-bond analysis.

## 3. Materials and Methods

### 3.1. Apparatus and General Information

The NMR (300 MHz for ^1^H NMR, 75 MHz for ^13^C {^1^H} NMR) spectra were recorded on Bruker Avance 300 machine (Rheinstetten, Germany. The chemical shifts δ were reported in ppm relative to tetramethylsilane (TMS). Data were described as follows: chemical shift (δ in ppm), multiplicity (s = singlet, d = doublet, m = multiplet, q = quartet), coupling constants (Hz), integration. Optical rotations were determined by a Perkin Elmer polarimeter. Elemental analyses were performed on a Perkin Elmer 2400 Series II Elemental CHNS analyzer (Waltham, MA, USA). Thin-layer chromatography (TLC) was performed on silica gel plates (Merck, silica gel 60 F_254_ 0.2 mm, 200_200 nm) (Darmstadt, Germany) using UV light at 254 nm.

### 3.2. General Procedure for the Synthesis of (E,E)-3,5-bisarylidene-N-[(S)-(-)-methylbenzyl]-4-piperidones 1a–e

A mixture of *N*-[(*S*)-(-)-methylbenzyl]-4-piperidone **7** (2 mmol) and aromatic aldehydes **8** (4 mmol) in alkaline medium (30 mL NaOH (30%)) was stirred at room temperature for 30 min. The obtained solid was filtered and crystallized from methanol to give dipolarohiles **1a**–**e** in excellent yields (81–93%).

### 3.3. General Procedure for the Synthesis of Spiroxindolopyrrolidine-Piperidones 4 

A mixture of *(E,E)-*3,5-bisarylidene-*N*-[(*S*)-(-)-methylbenzyl]-4-piperidones **1a**–**e** (1 mmol), isatin **2** (1 mmol), glycine aminoester **3** or **3′** (1 mmol), and Et3N (1 mmol) in acetonitrile (10 mL) was carried out in acetonitrile at 80 °C for 2 h in the presence of triethylamine. After completion of the reaction (TLC monitoring), the solvent was removed under vacuum. The crude residue was chromatographed on silica gel (ethylacetate-cyclohexane = 7:3) to give the enantiopure spiroxindolopyrrolidines **4**.

### 3.4. Crystal Structure Determination

Data collection was performed on a Bruker D8 Venture four-circle diffractometer from Bruker AXS GmbH. CPAD detector used: Photon II from Bruker AXS GmbH; X-ray sources: Microfocus source IμS and microfocus source IμS Mo and Cu, respectively, from Incoatec GmbH with mirror opticsHELIOS and single-hole collimator from Bruker AXS GmbH.

Programs used for data collection: APEX4 Suite [57] (v2021.10-0) and integrated programs SAINT (V8.40A; integration) and SADABS (2018/7; absorption correction) from Bruker AXS GmbH [57]. The SHELX programs were used for further processing [58]. The solution of the crystal structures was done with the help of the program SHELXT [59], the structure refinement with SHELXL [60]. The processing and finalization of the crystal structure data was done with program OLEX2 v1.5 [61]. All non-hydrogen atoms were refined anisotropically. For the hydrogen atoms, the standard values of the SHELXL program were used with U_iso_ (H) = –1.2 U_eq_(C) for CH_2_ and CH and with U_iso_(H) = –1.5 U_eq_(C) for CH_3_. All H atoms were refined freely using independent values for each U_iso_(H).

Crystal data for C_41_H_41_N_3_O_6_, M = 671.77 g∙mol^−1^, dark orange crystals, crystal size 0.781 × 0.534 × 0.378 mm^3^, monoclinic, space group P2_1_, a = 11.6919(4) Å, b = 22.6612(7) Å, c = 13.0938(4) Å), α = 90°, β = 90.3920(10)°, γ = 90; V = 3469.15(19) Å^3^, Z = 4, D_calc_ = 1.286 g/cm^3^, T = 100 K, R_1_ = 0.0296, wR_2_ = 0.0749 for 45555 reflections with I > = 2σ (I) and 14594 independent reflections. Largest diff. peak/hole/e Å^−3^ 0.14/-0.17. Flack parameter 0.00(7). Data were collected using graphite monochromated CuK_α_ radiation λ = 1.54178 Å and have been deposited at the Cambridge Crystallographic Data Centre as CCDC 2170410. (Supplementary Materials). The data can be obtained free of charge from the Cambridge Crystallographic Data Centre via http://www.ccdc.cam.ac.uk/getstructures. 

### 3.5. Molecular Modelling

#### 3.5.1. Molecular Docking

The crystal structure of SARS-CoV-2 M^pro^ complexed with the reversible inhibitor “X77” (PDB code: 6W63) [50] was used to conduct docking experiments on GOLD (version 5.8) [51,52] as previously described [53]. All figures were prepared using PyMol [62].

#### 3.5.2. Molecular Dynamics Simulations

MD simulations were done utilizing the PMEMD.cuda code of the AMBER Molecular Dynamics package [54] following the same previously described protocol of minimization, heating, density equilibration and production [53]. Each system was heated for 20 ps followed by density equilibrations for 200 ps and finally the production run was for 80 ns. All simulations were run on an Intel core i7 @2.60 GHz workstation with Nvidia GeForce GTX 1660 Ti GPU with an average CPU time of 100 ns/day. Analysis of the trajectories were done using CPPTraj [63], VMD [64] was used to visually examine the trajectories and RMSD Plots were generated using XMgrace [65].

Approximately 15192 TIP3P water molecules were used to solvate each ligand–M^pro^ complex within a cubic box of dimensions 91.3 × 69.7 × 95.4 Å box maintaining at least 12 Å between the box boundaries and the closest solute atoms. To neutralize the solvated systems, 27 NaCl counter ions were added to reach a salt concentration of 100 mM. Two minimization cycles were conducted on each complex to remove bad contacts where strong restraints of 500 kcal/mol.Å^2^ were applied on the non-solvent residues during the first minimization run. The system was then heated slowly from 0 K to 300 K using the Langevin thermostat. The density equilibrations and production runs were performed at constant pressure (1 atm) and temperature held constant by applying Langevin dynamics [66]. Long-range electrostatics were treated using the Particle-Mesh Ewald method [67] under periodic boundary conditions. 

#### 3.5.3. Free Energy Calculations

The MM-GBSA method [56] was used to compute the binding free energy using the MMPBSA.py script [68] included in AmberTools [54]. The calculations were conducted for the last 10 ns of the MD trajectory with snapshots sampled at a regular interval of 10 ps ending up with a total of 1000 frames. The MMGBSA estimates the binding free energy (ΔG_bind_) as, ΔG =ΔE_MM_ + ΔG_sol_—TΔS, where ΔE_MM_ accounts for the bonding, electrostatic and van der Waals interactions as in standard molecular mechanics. The solvation energy, ΔG_solv_, is the sum of the polar and non-polar contributions of solvation, with the former calculated using a Generalized-Born model while the latter contributions are computed based on the size of the solvent-accessible surface area in the ligand and M^pro^. The (TΔS) term corresponds to conformational entropy which was not included in energy calculations as it is computationally expensive.

## 4. Conclusions

To sum up, an efficient method for the asymmetric synthesis of a small library of enantiomerically pure spirooxindolopyrrolidine-grafted piperidones in good yields and high diastereoselectivity was developed. These spiroheterocycles were synthesized by 1,3-dipolar cycloadditions of various stabilized azomethineylides with chiral dipolarophiles derived from *N*-[(*S*)-(-)-methylbenzyl]-4-piperidone. Docking and molecular dynamics simulation studies showed three of these compounds exhibiting the same binding mode to SARS-CoV-2 M^pro^, binding to the key subsites with high stability. Post-MD simulation analyses showed a slight superiority of compound **4b** in terms of H-bonds stability and free-binding energy. Our findings suggest these spirooxindolopyrrolidine-piperidones as promising leads for further lead optimization and development as SARS-CoV-2 M^pro^ inhibitors.

## Figures and Tables

**Table 1:** Analysis of the H-bond interactions throughout the 80 ns MD trajectory of compounds **4a**, **4b** and **4e**, displaying only H-bonds that were maintained in more than 10% of the trajectory snapshots. For each H-bond, column 1 represents the acceptor residue and atom name. Columns 2 and 3 indicate the name of the H-atom and the electronegative atom attached to it on the donor residue, respectively. Interactions with residues of S1 and S2 subsites are shown in blue and red, respectively.

HB-Acceptor	HB-Donor	Donor	% *	Average H-bond	
				Distance (Å)	Angle
Compound **4a**					
HIS_164@O	LIG_307@H2	LIG_307@N2	45%	2.78	157.1
LIG_307@O6	GLY_143@H	GLY_143@N	16%	2.88	149.0
LIG_307@O2	ASN_142@HD22	ASN_142@ND2	12%	2.84	157.2
Compound **4b**					
LIG_307@O1	HIS_41@HE2	HIS_41@NE2	78%	2.76	154.3
HIS_164@O	LIG_307@H2	LIG_307@N2	62%	2.82	154.6
LIG_307@O6	GLY_143@H	GLY_143@N	43%	2.85	149.0
LIG_307@O2	ASN_142@HD21	ASN_142@ND2	21%	2.92	157.2
Compound **4e**					
LIG_307@O2	ASN_142@HD22	ASN_142@ND2	32%	2.88	158.9
HIS_164@O	LIG_307@H2	LIG_307@N2	19%	2.8525	156.1

* Percentage of persistence along the MD trajectory.

**Table 2:** List of MM-GBSA binding free energies (in kcal/mol) for compounds **4a**, **4b** and **4e** bound to SARS-CoV-2 main protease.

Compound	MMGBSA (kcal/mol)	Std Deviation	Std. Error of Mean
**4a**	−30.7	7.7	1.4
**4b**	−35.2	8.2	1.5
**4e**	−30.4	7.1	0.7
**Control “X77”** [53]	−39.5	3.2	0.1

## Data Availability

The data presented in this study are not available from the authors.

## Supplementary Materials

The following supporting information can be downloaded at: https://www.mdpi.com/article/10.3390/molecules27123945/s1, compound characterization data of dipolarophiles **1a**–**e** and spirooxindolopyrrolidines **4a**–**f**. Copies of ^1^H and ^13^C {^1^H} NMR spectra of dipolarophiles **1a**–**e** and spirooxindolopyrrolidines **4a**–**f** (Figures S1–S27) and crystallographic data of **4c** (Tables S1 and S2).
