# Supplementary material for: Design of Novel Enantiopure Dispirooxindolopyrrolidine-Piperidones as Promising Candidates toward COVID-19: Asymmetric Synthesis, Crystal Structure and In Silico Studies
Source: Molecules. 2022 Jun 20;27(12):3945. doi: 10.3390/molecules27123945 (PMC9228936; doi:10.3390/molecules27123945)
Supplement: Supplementary file 1 [file molecules-27-03945-s001.zip › molecules-1768813-supplementary.pdf]

molecules

Supplementary Materials

Design of novel enantiopure dispirooxindolopyrrolidine-piperidones as promising candidates toward COVID-19: Asymmetric synthesis, crystal structure and *in silico* studies

Amani Toumi [1], Sarra Boudriga [1,*], Yasmine M. Mandour [2], Ahmed A. Mekki [2], Michael Knorr [3], Carsten Strohmann [4], Jan-Lukas Kirchhoff [4], Mansour Sobeh [5,*]

[1] Laboratory of Heterocyclic Chemistry Natural Product and Reactivity (LR11ES39), Department of Chemistry, Faculty of Science of Monastir, University of Monastir, Monastir 5019, Tunisia; amanitoumi45@gmail.com (A.T.); sarra.boudriga@fsm.rnu.tn (S.B.)
[2] School of Life and Medical Sciences, University of Hertfordshire hosted by Global Academic Foundation, New Administrative Capital, Cairo, Egypt; yasminemandour@yahoo.com (Y.M.M.); ahmed.abdelrasool@gaf.ac (A.A.M.)
[3] Institut UTINAM-UMR CNRS 6213, Université Bourgogne Franche-Comté, 16 Route de Gray, 25030 Besançon, France ; michael.knorr@univ-fcomte.fr (M.K.)
[4] Faculty of Chemistry, Inorganic Chemistry, Technische Universität Dortmund, Otto-Hahn-Strasse 6, 44227 Dortmund, Germany; carsten.strohmann@tu-dortmund.de (C.S.); jan-lukas.kirchhoff@tu-dortmund.de (J.-L.K.)
[5] AgroBioSciences Research, Mohammed VI Polytechnic University, Lot 660–Hay MoulayRachid, Ben Guerir 43150, Morocco; mansour.sobeh@um6p.ma
* Correspondence: sarra.boudriga@fsm.rnu.tn (S.B.); mansour.sobeh@um6p.ma (M.S.)

Table of contents

Page

1. Characterization data of compounds **1a-e**
S2
2. Characterization data of compounds **4a-f**
S2-S3
3. Copies of ^{1}H- and ^{13}C-NMR spectra of compounds **1a-e** and **4a-f**
S4-S17
4. **Table S1**. Selected bond lengths (Å) and angles for compound **5c**.
S18
5. **Table S2**. Hydrogen Bonds for **5c**
S18

1. **Characterization data of compounds 1a-e**

(*E*,*E*)-3,5-bisbenzylidene-*N*-[(*S*)-(-)-methylbenzyl]-4-piperidone 1a.

Pale yellow solid; Yield: (644 mg, 85%); mp (°C ± 2) = 113 °C; $[\alpha]_D^{25}$ = -10.5 (c 0.20, $CHCl_3$); ^{1}H NMR (300 MHz, $CDCl_3$) δ_H: 1.35 (d, 3H, *J* = 6.6 Hz), 3.75 (q, 1H, *J* = 6.6 Hz), 3.86 (d, 2H, *J* = 15.1 Hz), 3.95 (d, 2H, *J* = 15.1 Hz), 7.16–7.42 (m, 15H, Ar-H), 7.83 (s, 2H); ^{13}C NMR (75 MHz, CDCl3) δ_c: 19.3, 51.5, 62.1, 127.3, 127.4, 128.3, 128.5, 128.9, 130.2, 133.6, 135.3, 136.7, 142.4, 188.0; Anal. Calcd for $C_{27}H_{25}NO$: C, 85.45; H, 6.64; N, 3.69%.

***(E,E)*-3,5-bis(*p*-methylbenzylidene)-*N*-[(S)-(-)-methylbenzyl]-4-piperidone 1b**.

Yellow solid; Yield: (640 mg, 81%); mp (°C ± 2) = 104 °C; $[\alpha]_D^{25}$ = -13.0 (c 0.20, $CHCl_3$); ^{1}H NMR (300 MHz, $CDCl_3$) δ_H: 1.38 (d, 3H, *J* = 6.3 Hz), 2.39 (s, 3H), 2.41 (s, 3H), 3.72 (q, 1H, *J* = 6.6 Hz), 3.85 (d, 2H, *J* = 15.3 Hz), 3.92 (d, 2H, *J* = 15.3 Hz), 7.21–7.34 (m, 9H, Ar-H), 7.54 (d, 4H, *J* = 7.8 Hz), 7.77 (s, 2H); ^{13}C NMR (75 MHz, CDCl3) δ_c: 19.4, 21.1, 51.6, 62.1, 124.1, 127.0, 127.9, 128.8, 129.2, 129.8, 131.6, 140.5, 142.7, 188.2. Anal. Calcd for $C_{29}H_{29}NO$: C, 85.47; H, 7.17; N, 3.44%. Found: C, 85.51; H, 7.20; N, 3.43%.

***(E,E)*-3,5-bis(*p*-methoxylbenzylidene)-*N*-[(S)-(-)-methylbenzyl]-4-piperidone 1c**.

Yellow solid; Yield: (800 mg, 93%); mp (°C ± 2) = 109 °C; $[\alpha]_D^{25}$ = -17.0 (c 0.20, $CHCl_3$);; ^{1}H NMR (300 MHz, $CDCl_3$) δ_H: 1.38 (d, 3H, *J* = 6.6 Hz), 3.75-3.94 (m, 11H), 6.88-6.93 (m, 2H), 7.19–7.34 (m, 11H, Ar-H), 7.81 (s, 2H); ^{13}C NMR (75 MHz, CDCl3) δ_c: 19.4, 51.5, 62.2, 115.5, 115.8, 127.3, 127.3, 128.3, 131.4, 132.0, 132.1, 133.3, 135.4, 142.6, 188.3. Anal. Calcd for $C_{29}H_{29}NO_3$: C, 79.24; H, 6.65; N, 3.19%. Found: C, 79.22; H, 6.63; N, 3.22%.

(*E*,*E*)-3,5-bis(*p*-chlorobenzylidene)-*N*-[(*S*)-(-)-methylbenzyl]-4-piperidone 1d.

Yellow solid; Yield: (789 mg, 88%); mp (°C ± 2) = 140 °C; $[\alpha]_D^{25}$ = - 21.0 (c 0.20, $CHCl_3$); ^{1}H NMR (300 MHz, $CDCl_3$) δ_H: 1.38 (d, 3H, *J* = 6.6 Hz), 3.73 (q, 1H, *J* = 6.8 Hz), 3.80 (d, 2H, *J* = 15.1 Hz), 3.87 (d, 2H, *J* = 15.1 Hz), 7.21–7.31 (m, 8H, Ar-H), 7.33-7.36 (m, 5H, Ar-H), 7.74 (s, 2H); ^{13}C NMR (75 MHz, CDCl3) δ_c: 18.9, 50.9, 61.6, 122.8, 126.9, 127.9, 131.1, 131.3, 133.5, 134.9, 134.9, 141.8, 187.3. Anal. Calcd for $C_{27}H_{23}Cl_2NO$: C, 72.32; H, 5.17; N, 3.12%. Found: C, 72.30; H, 5.20; N, 3.15%.

(*E*,*E*)-3,5-bis(*p*-nitrobenzylidene)-*N*-[(S)-(-)-methylbenzyl]-4-piperidone 1e.

Yellow solid; Yield: (0.78g, 85%); mp (°C ± 2) = 120°C; $[\alpha]_D^{25}$ = - 25.0 (c 0.20, $CHCl_3$); ^{1}H NMR (300 MHz, $CDCl_3$) δ_H: 1.34 (d, 3H, *J* = 6.6 Hz), 3.73 (q, 1H, *J* = 6.8 Hz), 3.80-3.91 (m, 4H), 7.14–7.32 (m, 7H, Ar-H), 7.40-7.44 (m, 4H, Ar-H), 8.21 (s, 2H), 8.22 (d, 2H, J = 8.7 Hz, Ar-H); ^{13}C NMR (75 MHz, CDCl3) δ_c: 18.7, 50.8, 61.8, 123.2, 126.8, 127.1, 128.0, 130.1, 133.7, 135.8, 140.9, 141.6, 147.0, 186.7. Anal. Calcd for $C_{27}H_{23}N_3O_5$: C, 69.07; H, 4.94; N, 8.95%. Found: C, 69.10; H, 4.91; N, 8.97%.

2. **Characterization data of compounds 4a-f**

Ethyl *(2R, 3R, 4S, 5S)*-3''-((*E*)-benzylidene)-2′,4''-dioxo-4-phenyl-1''-((*S*)-1-phenylethyl) dispiro [indoline-3′,2-pyrrolidine-3,5''-piperidine]-5-carboxylate (**4a**)

Yellow solid; Yield: (501 mg, 82%); mp (°C ± 2) = 175 °C; $[\alpha]_D^{25}$ = -157.0 (c 0.20, $CHCl_3$); ^{1}H NMR (300 MHz, DMSO-*d6*) δ_H: 1.04 (t, *J* = 6.9 Hz, 3H), 1.21 (d, *J* =6.6 Hz, 3H), 2.57-2.61 (m, 1H), 2.95-3.02 (m, 2H), 3.52-3.63 (m, 1H), 3.74-3.79 (m, 1H), 3.93-4.02 (m, 2H), 4.49 (d, *J* = 10.2 Hz, 1H), 4.97 (d, *J* = 10.5 Hz, 1H), 6.74–6.90 (m, 2H, Ar-H), 6.95–7.00 (m, 5H, Ar-H), 7.02–7.28 (m, 10H, Ar-H), 7.32–7.37 (m, 3H, Ar-H+ $1H_{ethylenic}$); ^{13}C NMR (75 MHz, DMSO-*d6*) δ_c: 13.9, 19.9, 50.1, 53.0, 54.0, 59.7, 62.0, 63.6, 64.4, 73.2, 121.1, 126.7, 126.8, 127.1, 127.4, 127.8, 127.9, 128.5, 128.6, 129.2, 130.2, 130.3, 130.4, 133.9, 134.1, 134.2, 135.8, 135.9, 136.7, 137.0, 140.7, 143.3, 172.4, 178.3, 196.9; Anal. Calcd for $C_{39}H_{37}N_3O_4$: C, 76.57; H, 6.10; N, 6.87%. Found: C, 76.60; H, 6.12; N, 6.83%.

Ethyl *(2R, 3R, 4S, 5S)*-3''-((*E*)-*p*-methylbenzylidene)-2′,4''-dioxo-4-(*p*-methylphenyl)-1''-((*S*)-1-phenylethyl) dispiro [indoline-3′,2-pyrrolidine-3,5''-piperidine]-5-carboxylate (**4b**)

Yellow solid; Yield: (492 mg, 77%); mp (°C ± 2) = 199 °C; $[\alpha]_D^{25}$ = -133.0 (c 0.20, $CHCl_3$); ^{1}H NMR (300 MHz, $CDCl_3$) δ_H: 1.09 (t, *J* = 6.9 Hz, 3H), 1.27 (d, *J* =7.8 Hz, 3H), 1.76 (d, *J* = 12.9 Hz, 1H), 2.33 (s, 3H), 2.37 (s, 3H), 2.71 (d, *J* = 14.4 Hz, 1H), 3.18-3.39 (m, 1H), 3.44-3.68 (m, 1H), 4.07-4.18 (m, 2H), 4.56 (d, *J* = 10.2 Hz, 1H), 4.96 (d, *J* = 10.2 Hz, 1H), 6.71–6.90 (m, 1H, Ar-H), 6.94–7.10 (m, 9H, Ar-H), 7.13–7.28 (m, 6H, Ar-H), 7.38–7.45 (m, 2H, Ar-H + $1H_{ethylenic}$), 7.96 (bs, 1H); ^{13}C NMR (75 MHz, CDCl3) δ_c: 13.5, 17.5, 20.6, 20.8, 49.7, 53.9, 55.3, 56.5, 60.4, 63.7, 65.9, 72.8, 108.6, 121.7, 121.8, 126.0, 126.1, 126.3, 126.5, 127.1, 127.2, 127.4, 128.3, 129.1, 129.6, 130.2, 131.7, 133.3, 136.1, 137.1, 138.7, 140.9, 171.6, 178.4, 198.8; Anal. Calcd. for $C_{41}H_{41}N_3O_4$: C, 76.97; H, 6.46; N, 6.57%. Found: C, 76.95; H, 6.49; N, 6.58%.

Ethyl *(2R, 3R, 4S, 5S)*-3''-((*E*)-*p*-methoxybenzylidene)-2′,4''-dioxo-4-(*p*-methoxyphenyl)-1''-((*S*)-1-phenylethyl) dispiro [indoline-3′,2-pyrrolidine-3,5''-piperidine]-5-carboxylate (**4c**)

dark orange crystals; Yield: (569 mg, 85%); mp (°C ± 2) = 168 °C; $[\alpha]_D^{25}$ = -125.0 (c 0.20, $CHCl_3$); ^{1}H NMR (300MHz, CDCl3) δ_H: 1.11 (t, *J* = 7.1 Hz, 3H), 1.25 (d, *J* = 6.6 Hz, 3H), 1.80 (d, *J* = 12.6 Hz, 1H), 2.37 (d, *J* = 12.6 Hz, 1H), 2.74 (d, *J* = 13.8 Hz, 1H), 3.16-3.20 (m, 1H), 3.34-3.62 (m, 1H), 3.78-3.83 (m, 6H), 4.05-4.24 (m, 2H), 4.53 (d, *J* = 10.2 Hz, 1H), 4.94 (d, *J* = 10.2 Hz, 1H), 6.69–7.09 (m, 10H, Ar-H), 7.12–7.16 (m, 6H, Ar-H), 7.19–7.28 (m, 2H, Ar-H), 7.44–7.51 (m, 2H, Ar-H + $1H_{ethylenic}$), 8.15 (bs, 1H); ^{13}C NMR (75 MHz, CDCl3) δ_c: 13.3, 18.7, 49.6, 51.6, 53.2, 54.4, 56.0, 60.4, 61.8, 63.5, 64.0, 73.0; 109.3, 113.4,

114.0, 120.9, 120.9, 125.4, 127.0, 127.9, 128.7, 129.3, 130.1, 130.6, 132.2, 136.7, 140.2, 141.2, 142.7, 158.2, 160.1, 171.8, 178.4, 198.7; Anal. Calcd for $C_{41}H_{41}N_3O_6$: C, 73.30; H, 6.15; N, 6.26%. Found: C, 73.28; H, 6.11; N, 6.28%.

Ethyl *(2R, 3R, 4S, 5S)*-3''-((*E*)-*p*-chlorobenzylidene)-2′,4''-dioxo-4-(*p*-chlorophenyl)-1''-((*S*)-1-phenylethyl) dispiro [indoline-3′,2-pyrrolidine-3,5''-piperidine]-5-carboxylate (4d)

Yellow solid; Yield: (511 mg, 75%); mp (°C ± 2) = 209°C; $[\alpha]_D^{25}$ = -110.0 (c 0.20, $CHCl_3$); ^1H NMR (300 MHz, DMSO-d_6) δ_H: 1.03 (t, *J* = 7.1 Hz, 3H), 1.18 (d, *J* = 6.6 Hz, 3H), 2.66 (d, *J* = 12.6 Hz, 1H), 3.00-3.24 (m, 2H), 3.27-3.28 (m, 1H), 3.48-3.57 (m, 1H), 3.98-4.07 (m, 2H), 4.46 (d, *J* = 10.5 Hz, 1H), 4.88 (d, *J* = 10.5 Hz, 1H), 6.70–6.79 (m, 1H, Ar-H), 6.82–6.93 (m, 2H, Ar-H), 6.94–7.19 (m, 15H, Ar-H), 7.30–7.35 (m, 1H); ^{13}C NMR (75 MHz, DMSO-d_6) δ_c: 13.9, 18.8, 49.3, 51.5, 54.2, 60.3, 61.5, 63.1, 64.2, 72.5, 109.0, 109.2, 127.2, 127.6, 127.7, 127.8, 128.7, 129.1, 129.3, 129.6, 130.1, 130.2, 131.6, 132.4, 134.3, 136.0, 139.1, 140.2, 142.7, 142.9, 171.6, 178.2, 198.6; Anal. Calcd for $C_{39}H_{35}Cl_2N_3O_4$: C, 68.82; H, 5.18; N, 6.17%. Found: C, 68.78; H, 5.20; N, 6.19%.

Ethyl *(2R, 3R, 4S, 5S)*-3''-((*E*)-*p*-nitrobenzylidene)-2′,4''-dioxo-4-(*p*-nitrophenyl)-1''-((*S*)-1-phenylethyl) dispiro [indoline-3′,2-pyrrolidine-3,5''-piperidine]-5-carboxylate (4e)

Yellow solid; Yield: (559 mg, 80%); mp (°C ± 2) = 192 °C; $[\alpha]_D^{25}$ = -117.0 (c 0.20, $CHCl_3$); ^1H NMR (300 MHz, $CDCl_3$) δ_H: 1.14 (t, *J* = 7.2 Hz, 3H), 1.27 (d, *J* = 6.6 Hz, 3H), 2.74 (t, *J* = 12.3 Hz, 1H), 3.00-3.23 (m, 2H), 3.35-3.37 (m, 1H), 3.55-3.58 (m, 1H), 4.06-4.22 (m, 2H), 4.77 (d, *J* = 9.6 Hz, 1H), 5.08 (d, *J* = 9.6 Hz, 1H), 6.74–6.93 (m, 4H, Ar-H), 7.04–7.24 (m, 7H, Ar-H), 7.42–7.59 (m, 1H, Ar-H), 7.61–7.64 (m, 2H), 8.04–8.10 (m, 2H), 8.14–8.18 (m, 2H); ^{13}C NMR (75 MHz, $CDCl_3$) δ_c: 13.6, 17.8, 52.6, 54.0, 60.8, 62.8, 63.5, 63.9, 64.7, 73.4, 109.7, 122.2, 122.8, 122.9, 123.1, 123.1, 126.5, 126.8, 127.7, 127.8, 129.0, 130.1, 130.1, 131.0, 134.4, 140.3, 141.2, 146.7, 147.0, 171.3, 178.1, 198.6; Anal. Calcd for $C_{39}H_{35}N_5O_8$: C, 66.75; H, 5.03; N, 9.98%. Found: C, 66.77; H, 5.05; N, 10.00%.

Methyl *(2R, 3R, 4S, 5S)*-3''-((*E*)-*p*-methylbenzylidene)-2′,4''-dioxo-4-(*p*-methylphenyl)-1''-((*S*)-1-phenylethyl) dispiro [indoline-3′,2-pyrrolidine-3,5''-piperidine]-5-carboxylate (4f)

Yellow solid; Yield: (474 mg, 72%); mp (°C ± 2) = 200 °C; $[\alpha]_D^{25}$ = -165.0 (c 0.20, $CHCl_3$); ^1H NMR (300 MHz, DMSO-d_6) δ_H: 1.07 (d, *J* = 6.6 Hz, 3H), 2.72 (d, *J* = 6.0 Hz, 1H), 3.16 (d, *J* = 12.6 Hz, 1H), 3.26 (d, *J* = 12.6 Hz, 1H), 3.49-3.51 (m, 1H), 3.64 (d, *J* = 12 Hz, 1H), 3.73 (s, 3H), 3.80 (s, 3H), 3.81 (s, 3H), 4.81 (d, *J* = 10.5 Hz, 1H), 4.95 (d, *J* = 10.5 Hz, 1H), 6.74–7.00 (m, 4H, Ar-H), 7.02–7.28 (m, 14H, Ar-H), 7.32–7.37 (m, 1H); ^{13}C NMR (75 MHz, DMSO-d_6) δ_c: 17.3, 49.0, 51.0, 53.5, 53.9, 54.8, 59.4, 61.6, 62.8, 63.4, 72.6, 108.4, 121.7, 121.8, 126.4, 126.6, 127.0, 127.2, 127.4, 127.7, 127.9, 129.2, 129.3, 129.4, 129.5, 132.8, 134.7, 136.1, 136.3, 137.1,137.3, 139.0, 141.4, 141.5, 171.8, 177.4, 197.4; Anal. Calcd for $C_{40}H_{39}N_3O_6$: C, 73.04; H, 5.98; N, 6.39%. Found: C, 73.06; H, 5.95; N, 6.42%.

3. Copies of ^{1}H- and ^{13}C-NMR spectra of compounds 1a-e and 4a-f

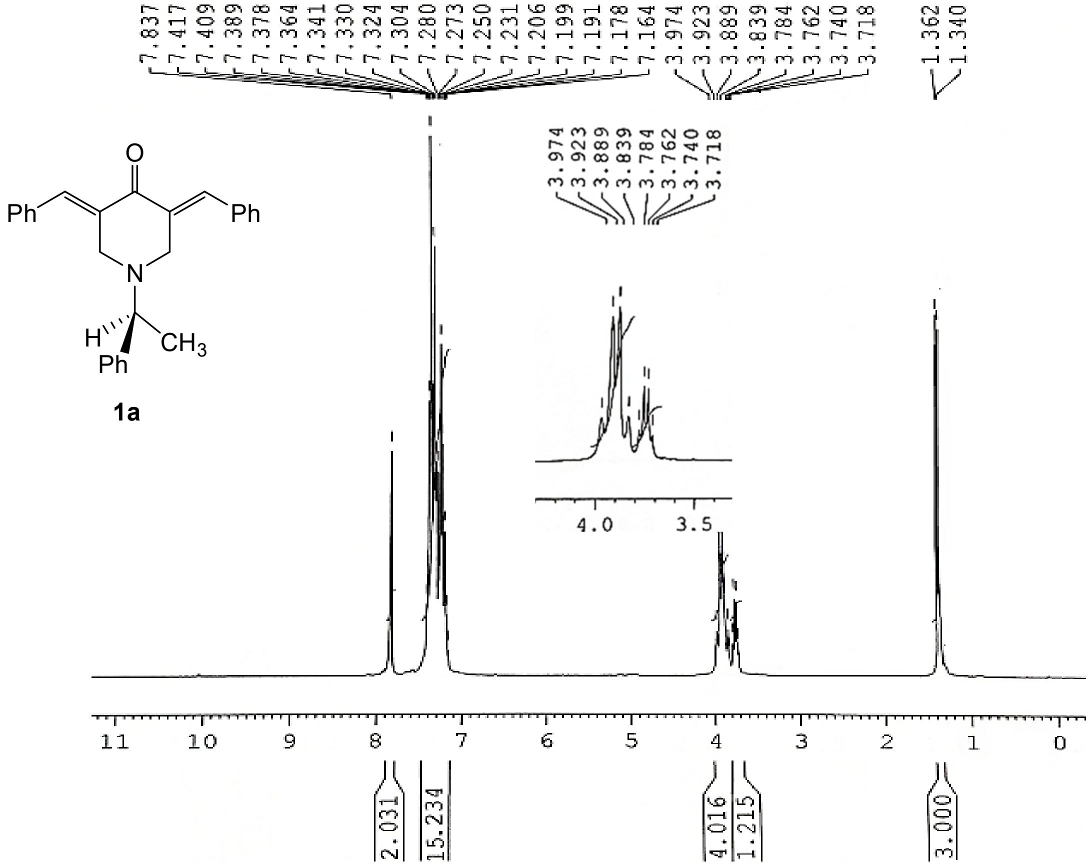

1a

Figure S1. ^{1}H NMR spectrum of compound **1a** (300 MHz, $CDCl_3$)

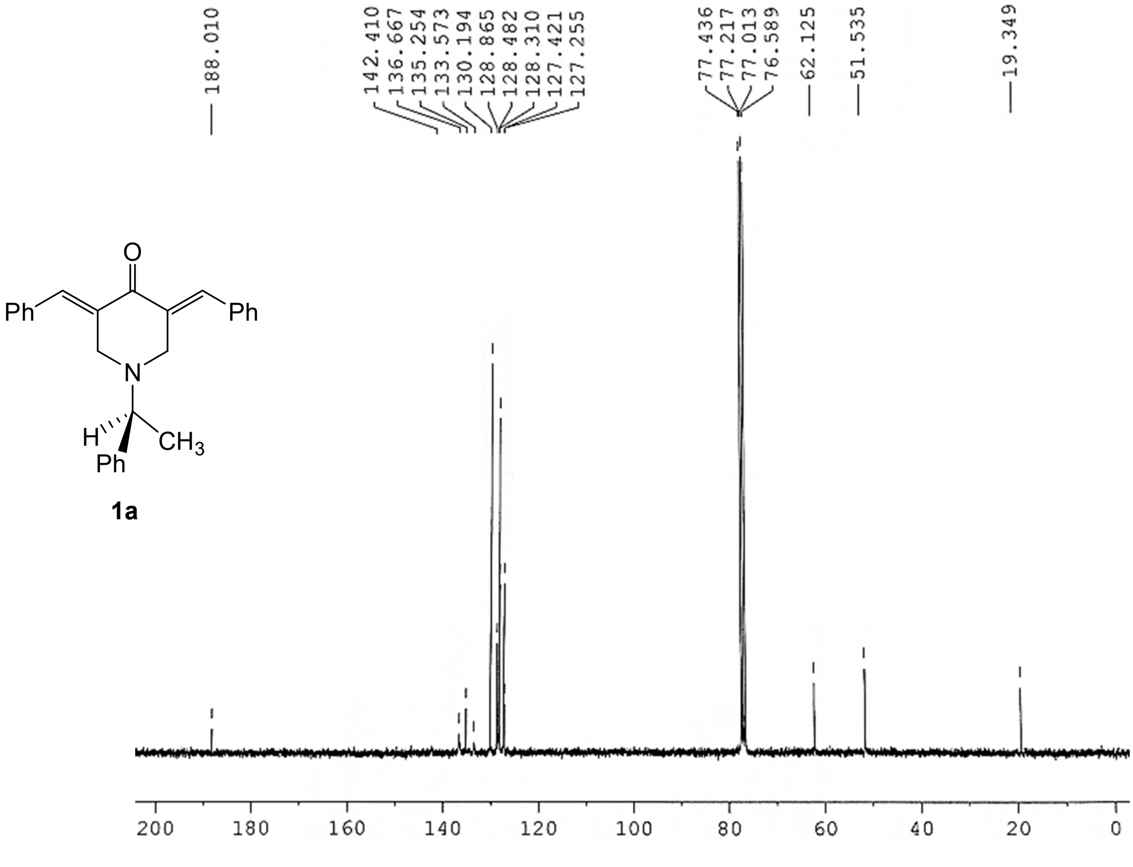

1a

Figure S2. $^{13}C\ \{^{1}H\}$ NMR spectrum of compound **1a** (75 MHz, $CDCl_3$)

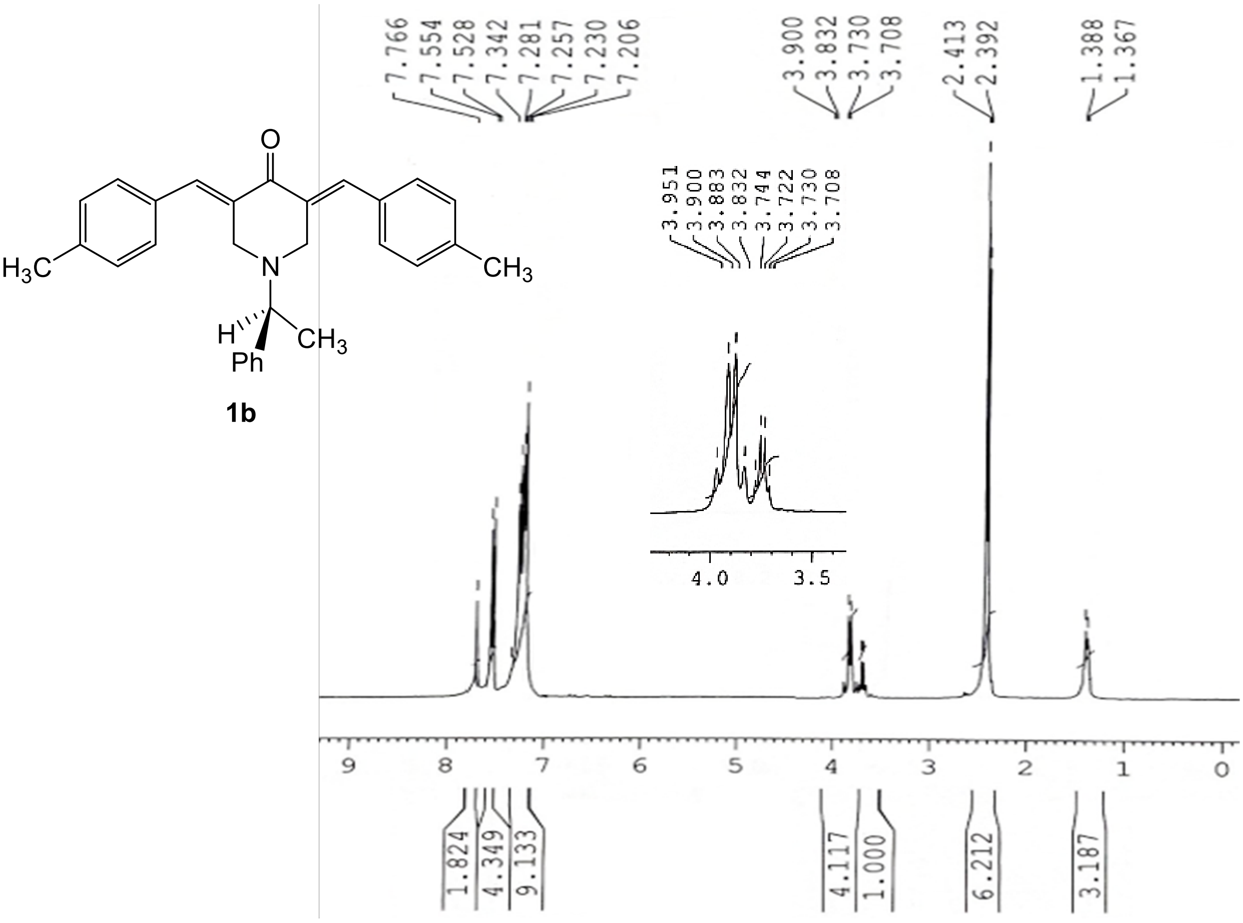

Figure S3. ^{1}H NMR spectrum of compound **1b** (300 MHz, $CDCl_3$)

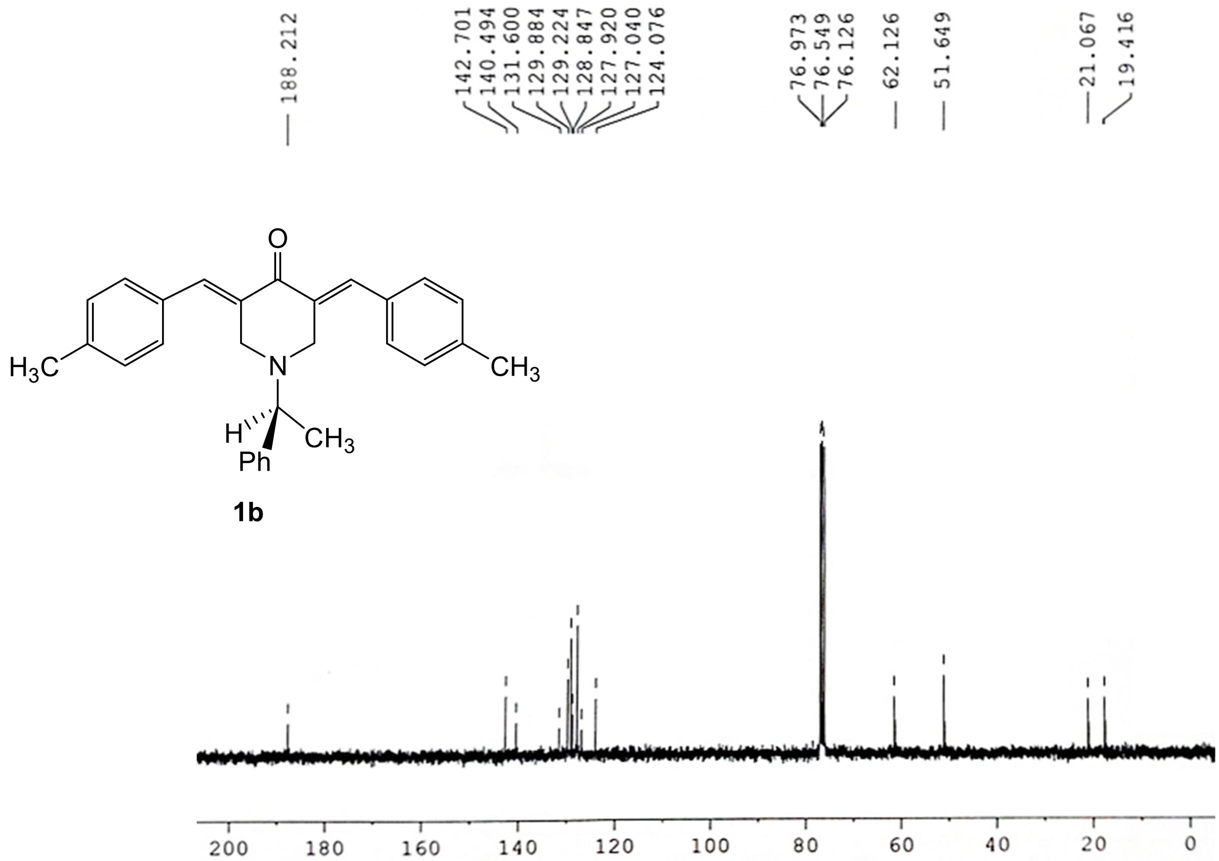

Figure S4. ^{13}C {^1H} NMR spectrum of compound **1b** (75 MHz, $CDCl_3$)

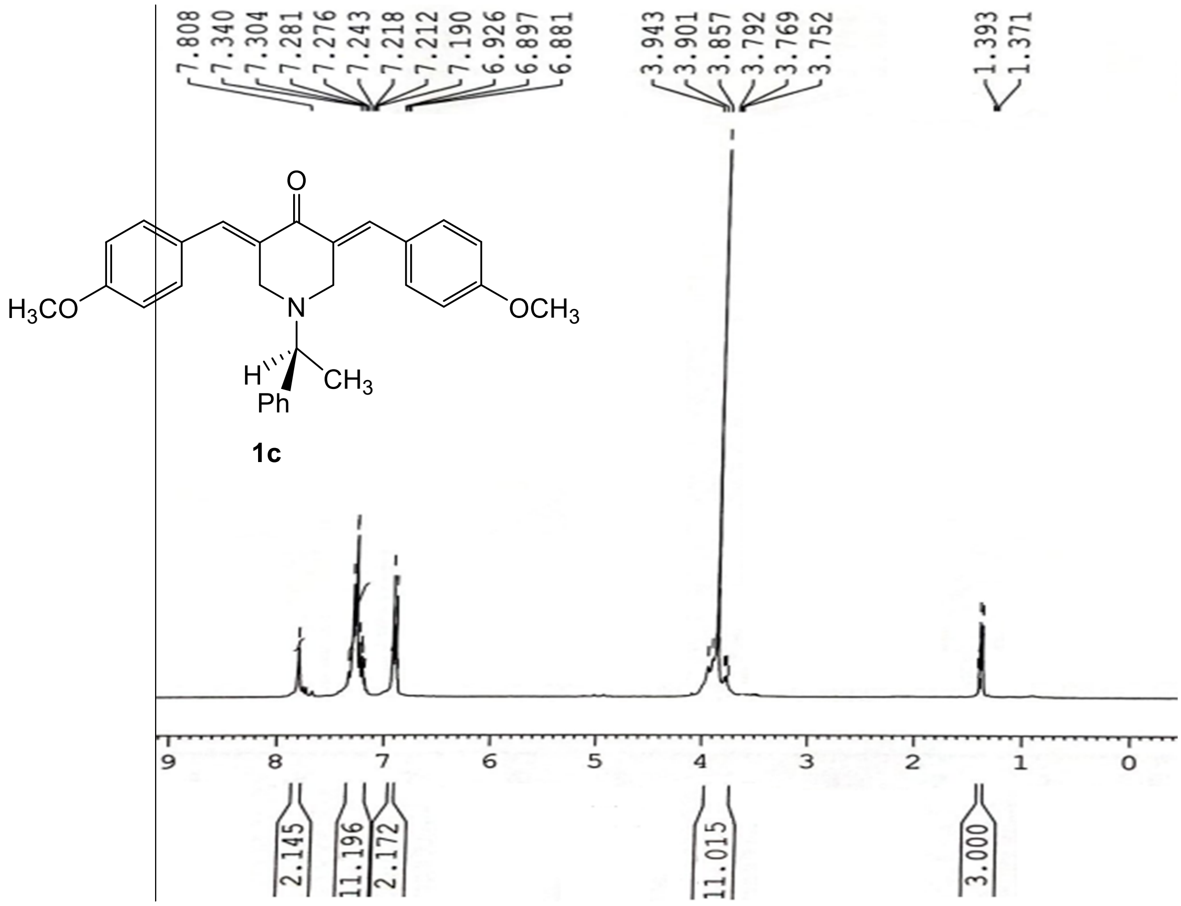

Figure S5. ^1H NMR spectrum of compound **1c** (300 MHz, $CDCl_3$)

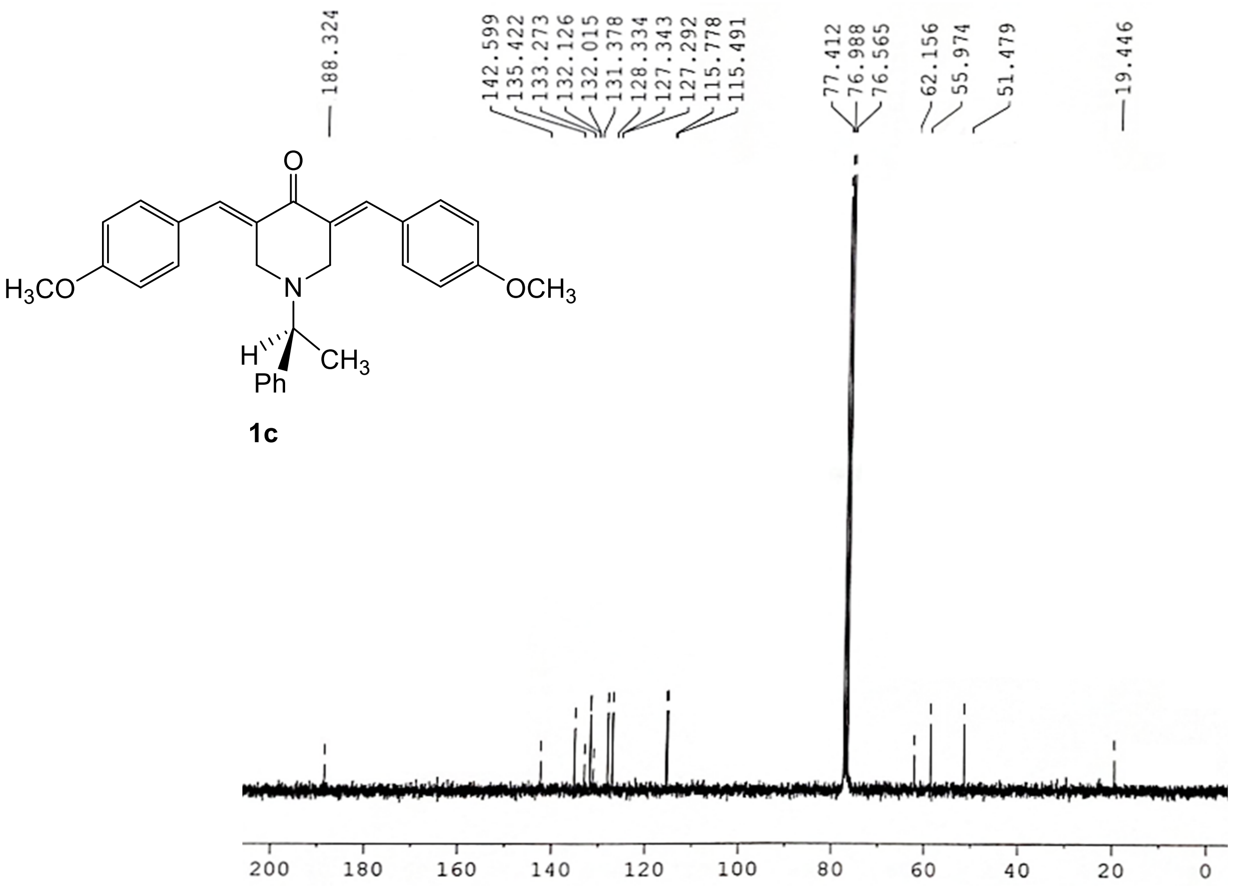

Figure S6. ^{13}C {^{1}H} NMR spectrum of compound **1c** (75 MHz, $CDCl_3$)

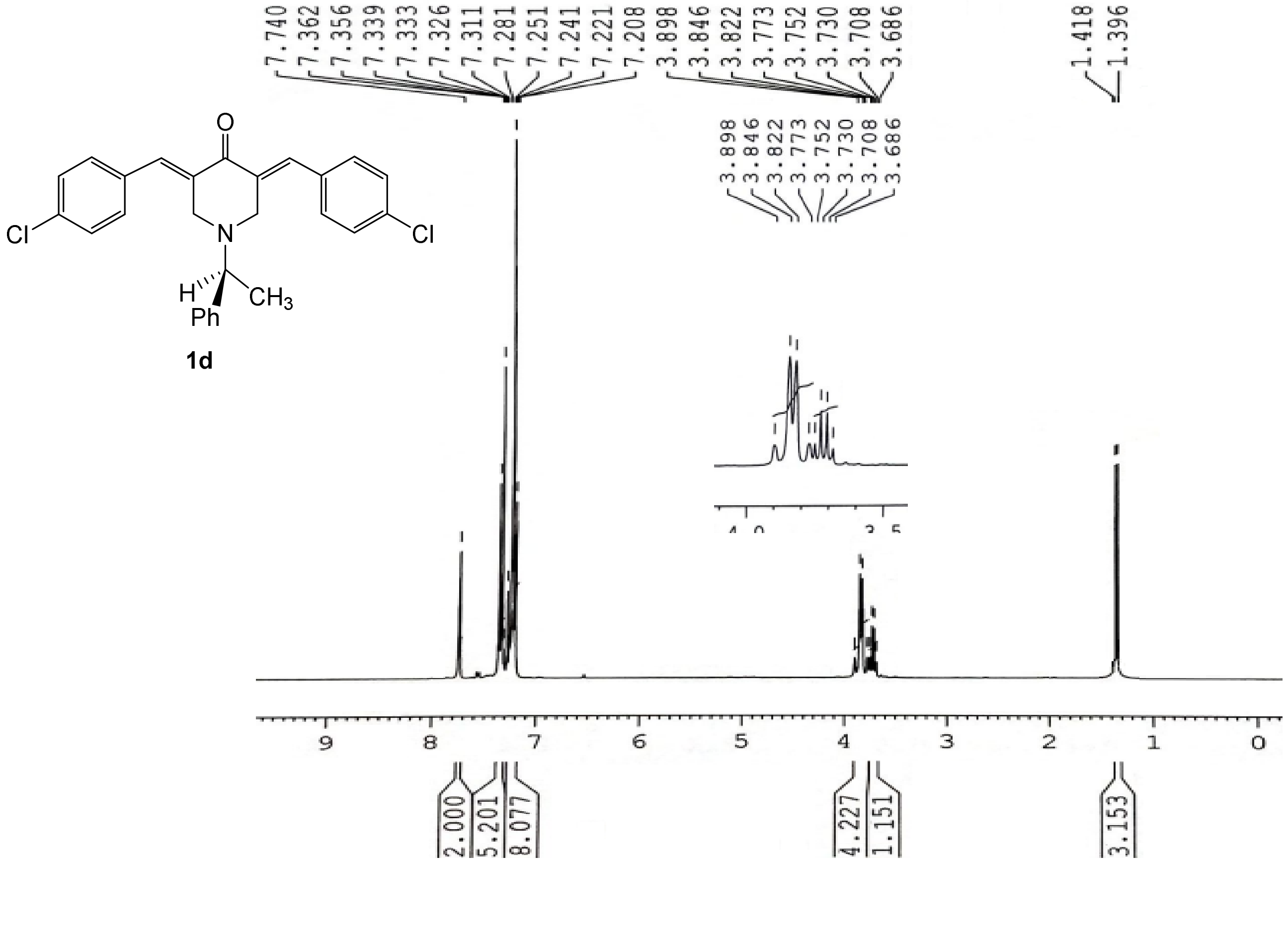

Figure S7. ^{1}H NMR spectrum of compound **1d** (300 MHz, $CDCl_3$)

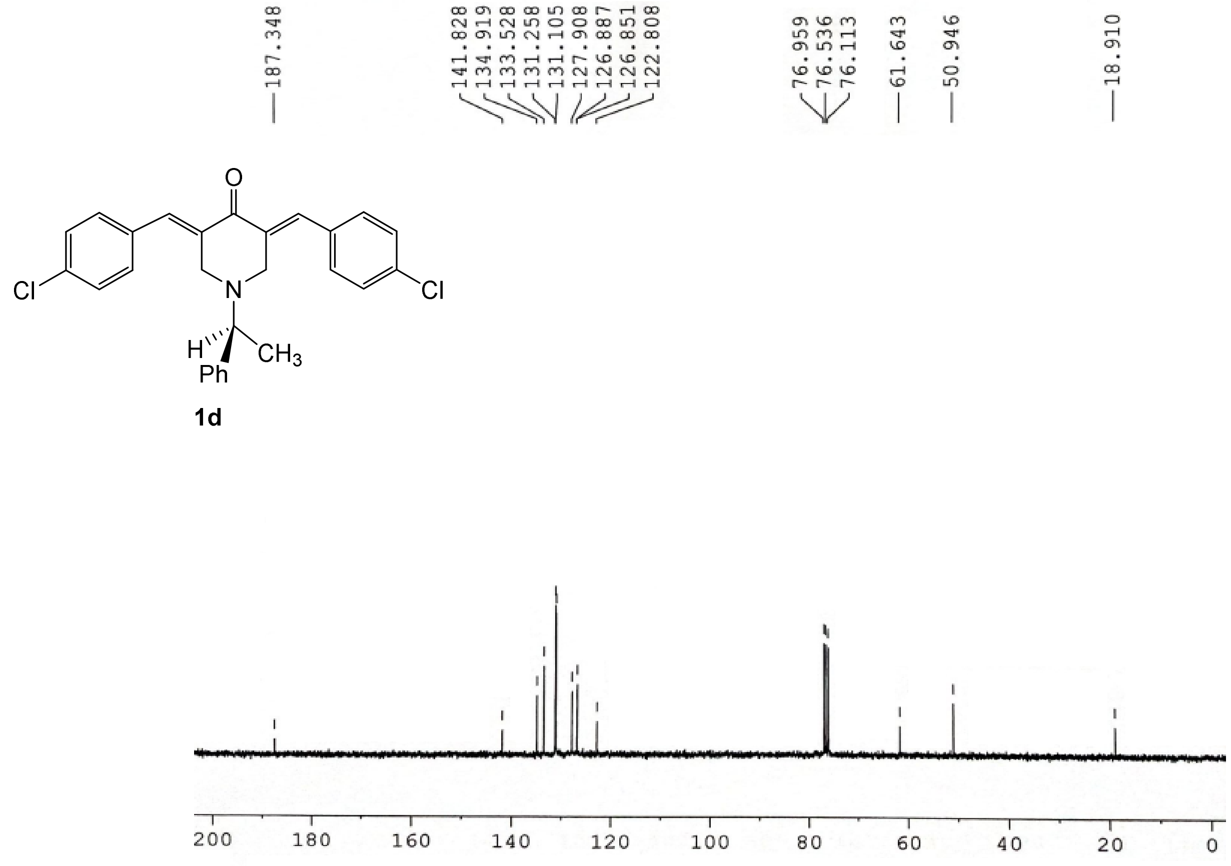

Figure S8. ^{13}C {^{1}H} NMR spectrum of compound **1d** (75 MHz, $CDCl_3$)

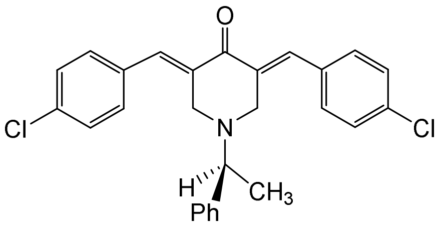

1d

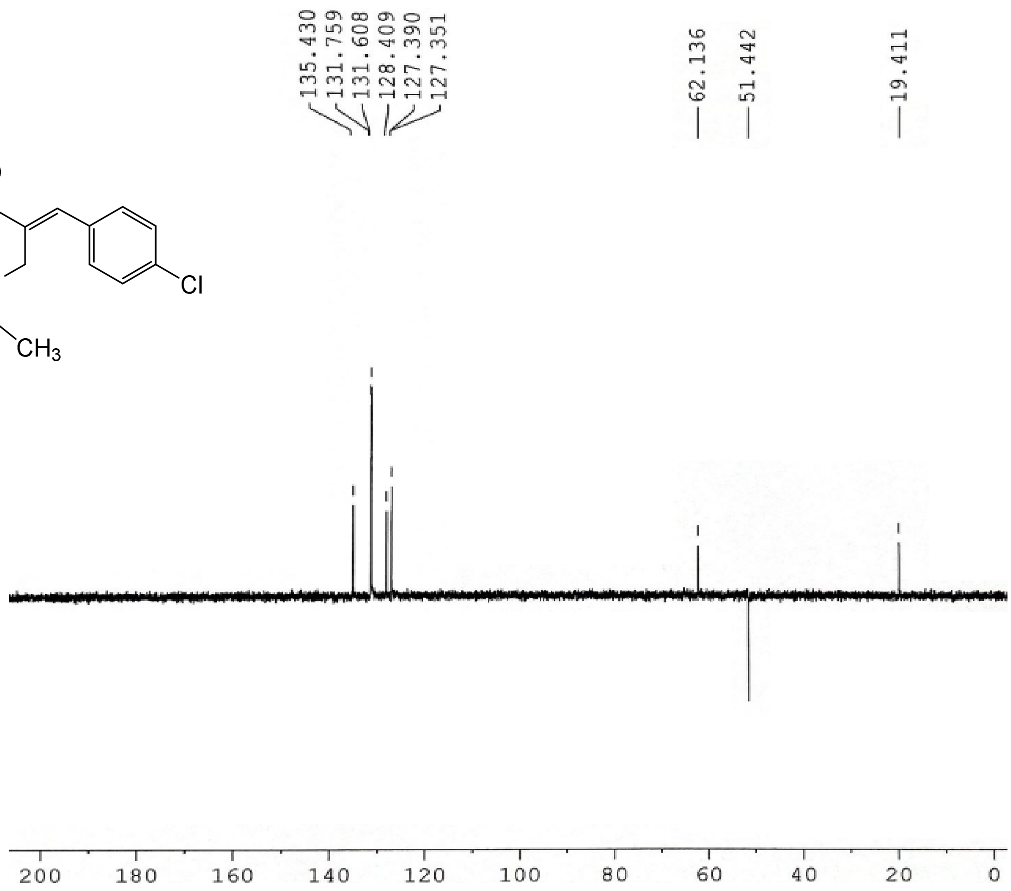

Figure S9. DEPT-135 spectrum of compound **1d** (75 MHz, $CDCl_3$)

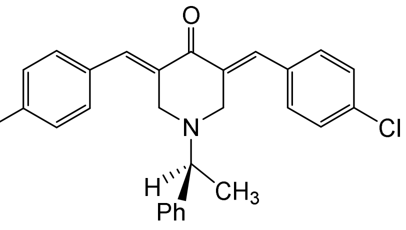

1d

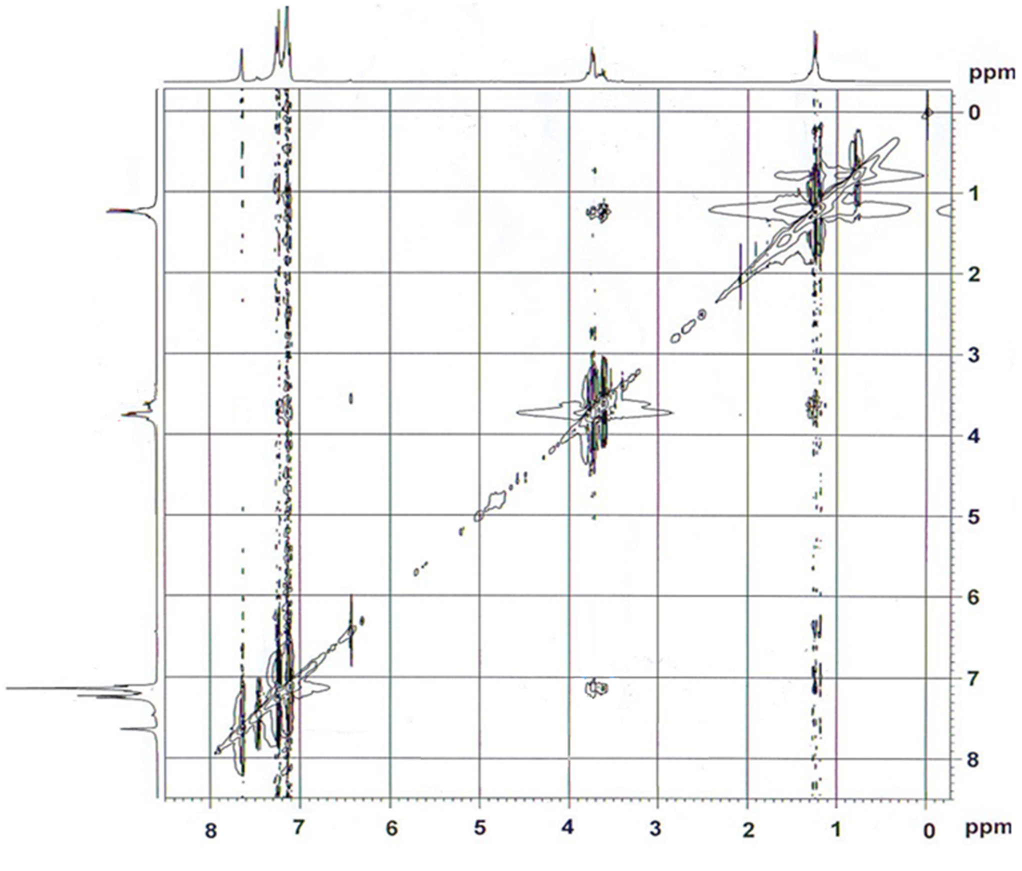

Figure S10. NOESY spectrum of compound **1d** (300 MHz, $CDCl_3$)

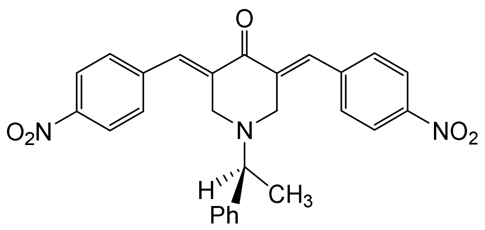

1e

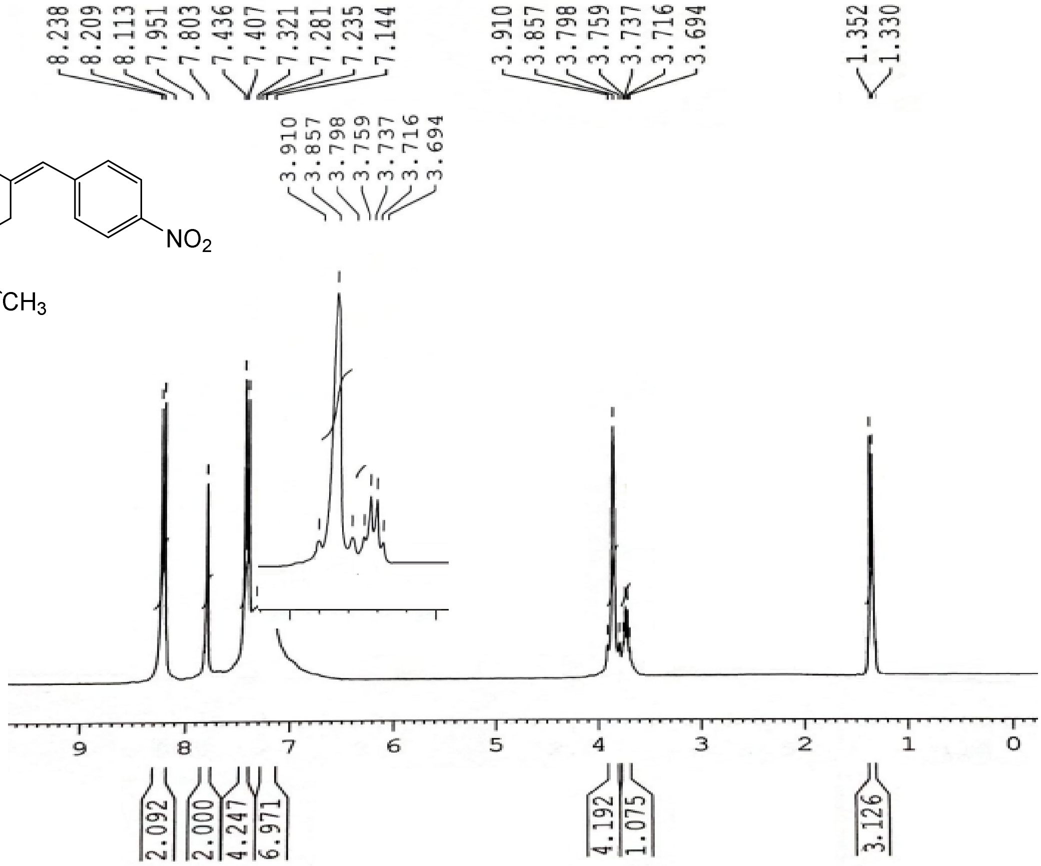

Figure S11. ^1H NMR spectrum of compound **1e** (300 MHz, $CDCl_3$)

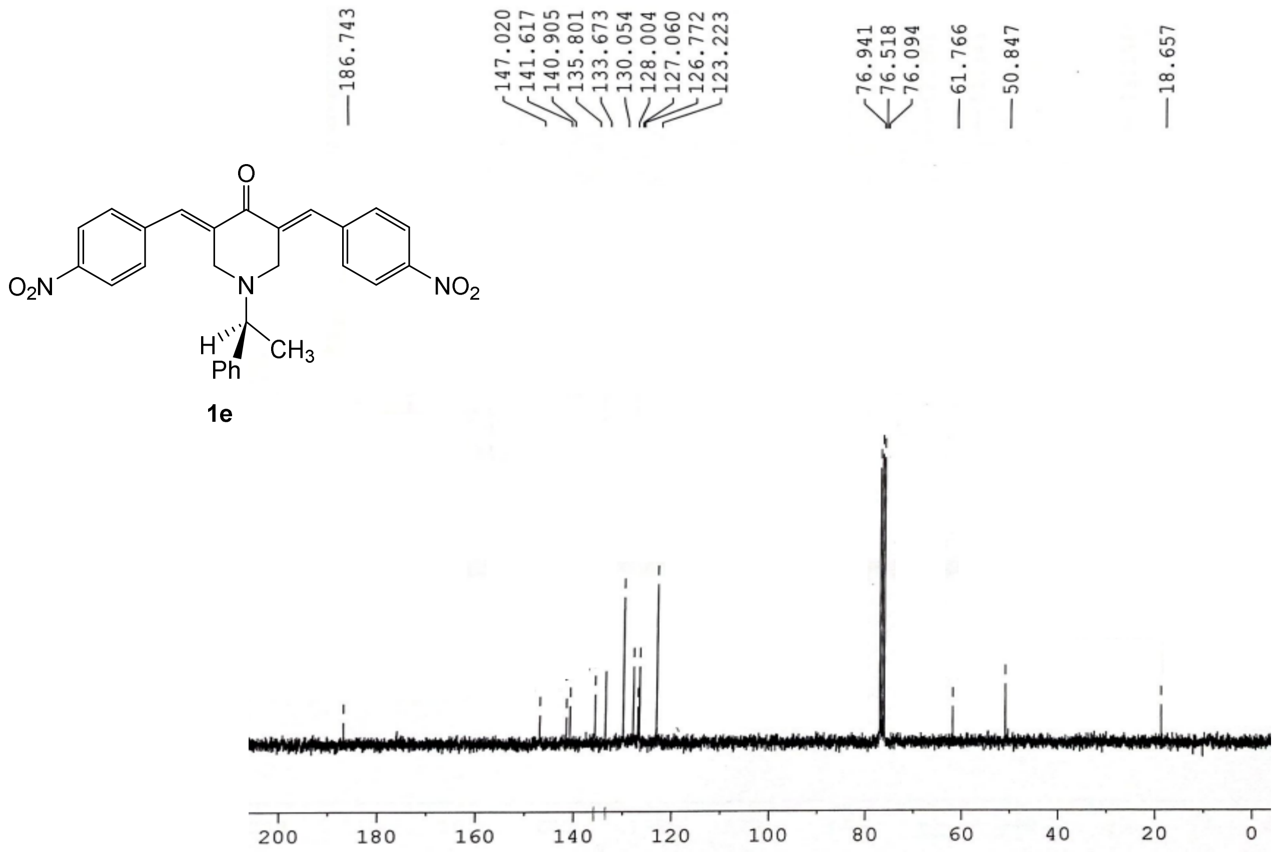

Figure S12. $^{13}C\{^1H\}$ NMR spectrum of compound **1e** (75 MHz, $CDCl_3$)

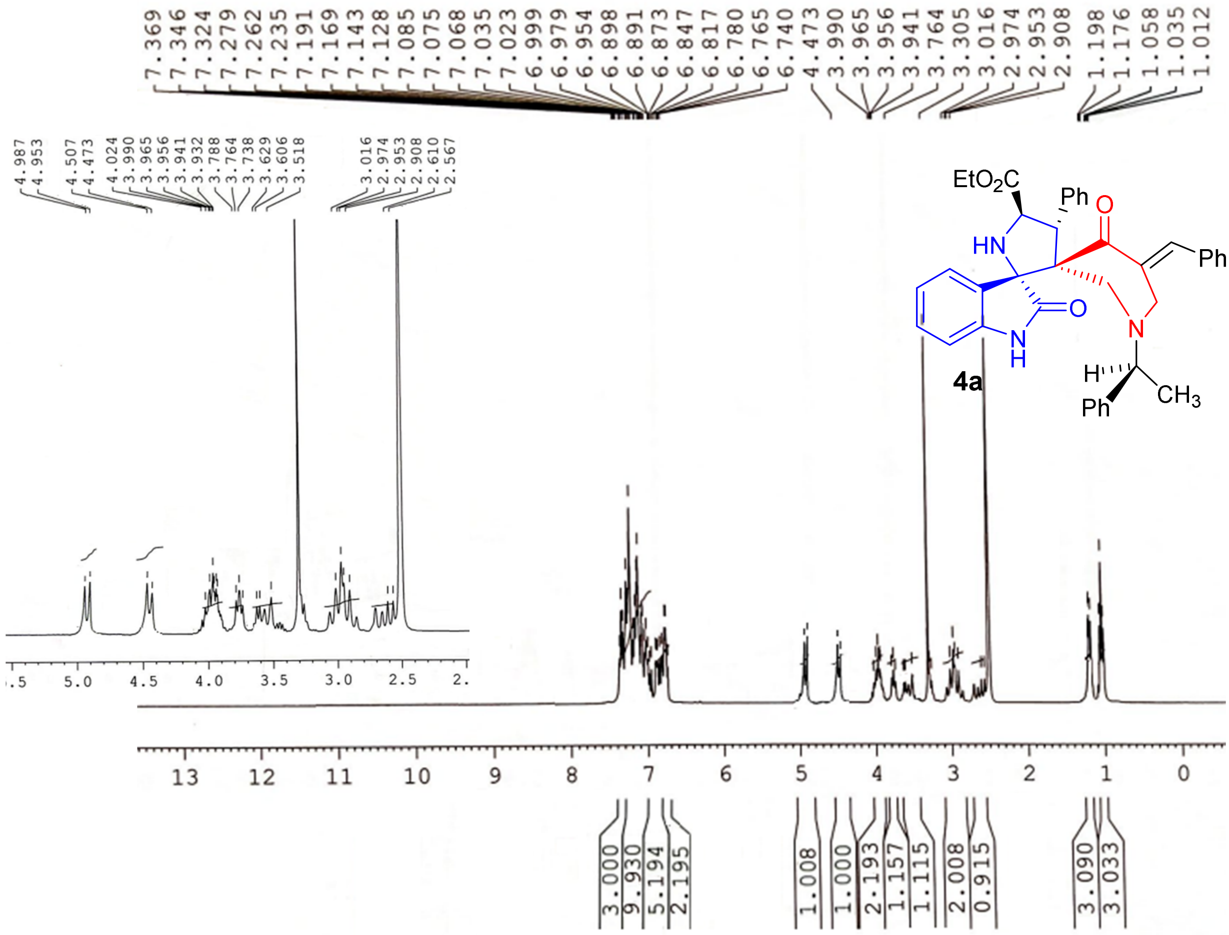

Figure S13. ^{1}H NMR spectrum of compound **4a** (300 MHz, DMSO-d_6)

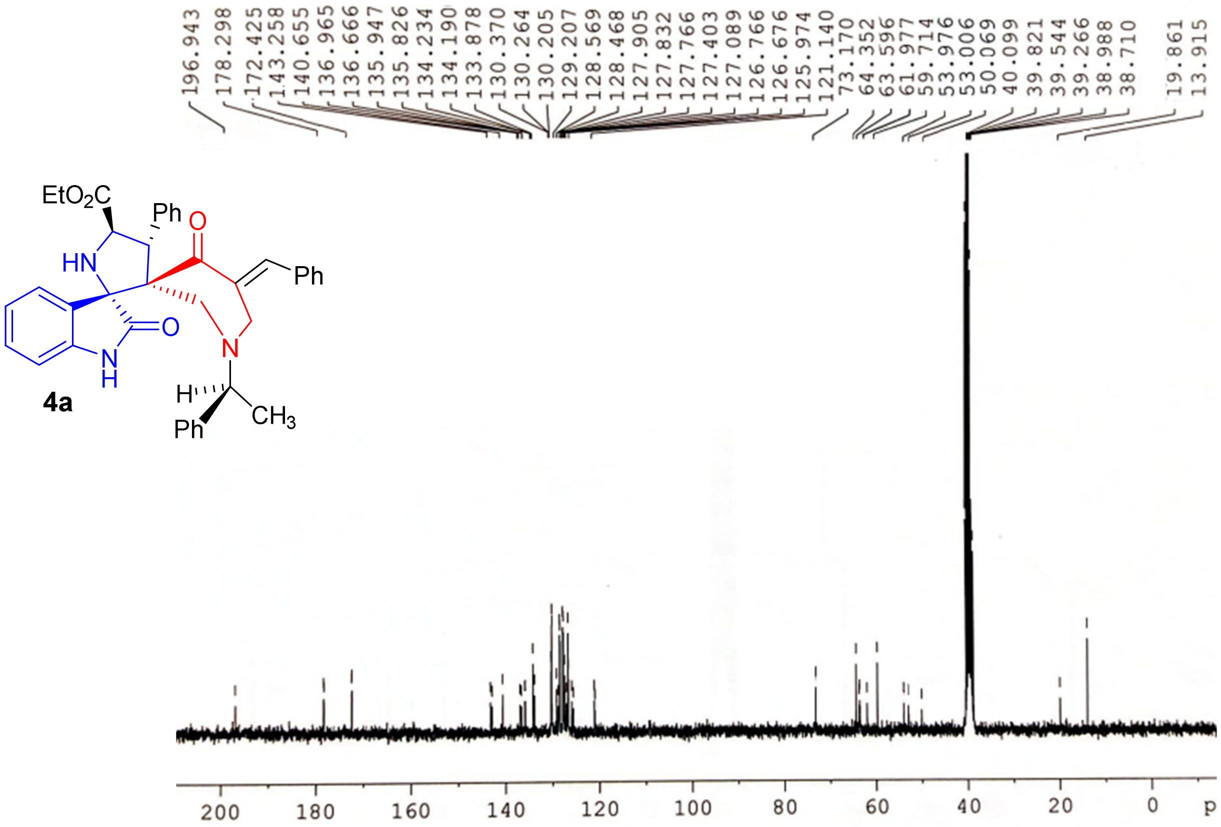

Figure S14. ^{13}C {^{1}H} NMR spectrum of compound **4a** (75 MHz, DMSO-d_6)

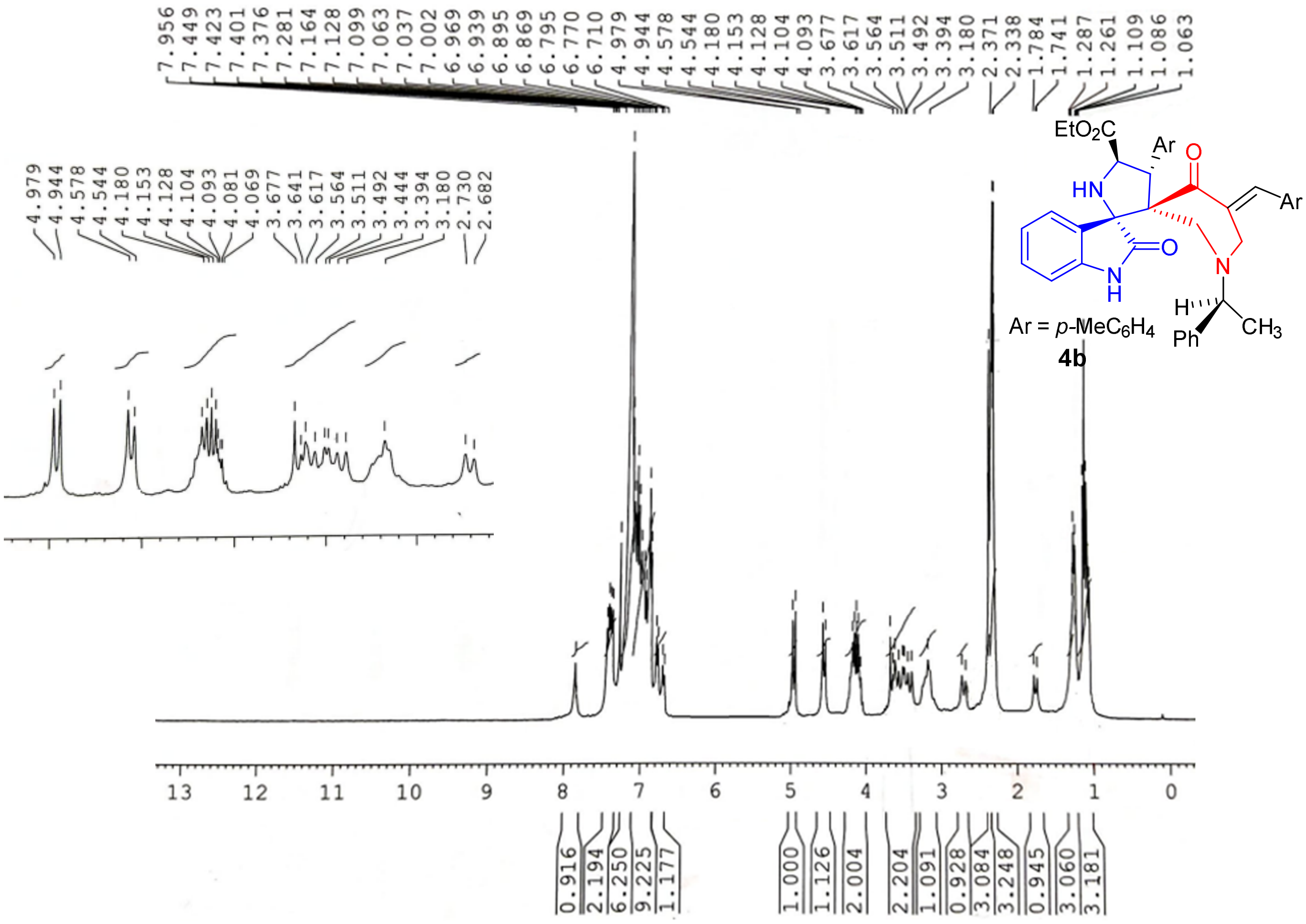

Figure S15. ^{1}H NMR spectrum of compound **4b** (300 MHz, $CDCl_3$)

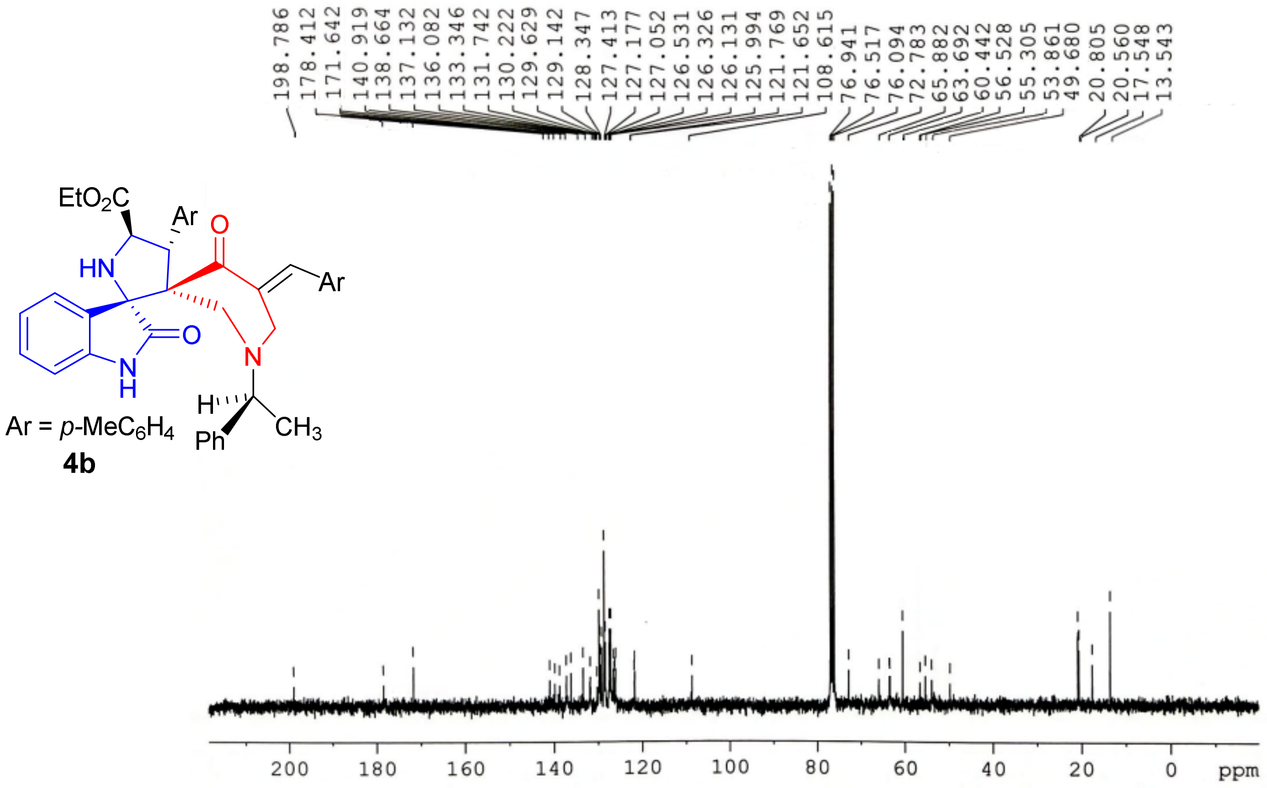

Figure S16. ^{13}C {^{1}H} NMR spectrum of compound **4b** (75 MHz, $CDCl_3$)

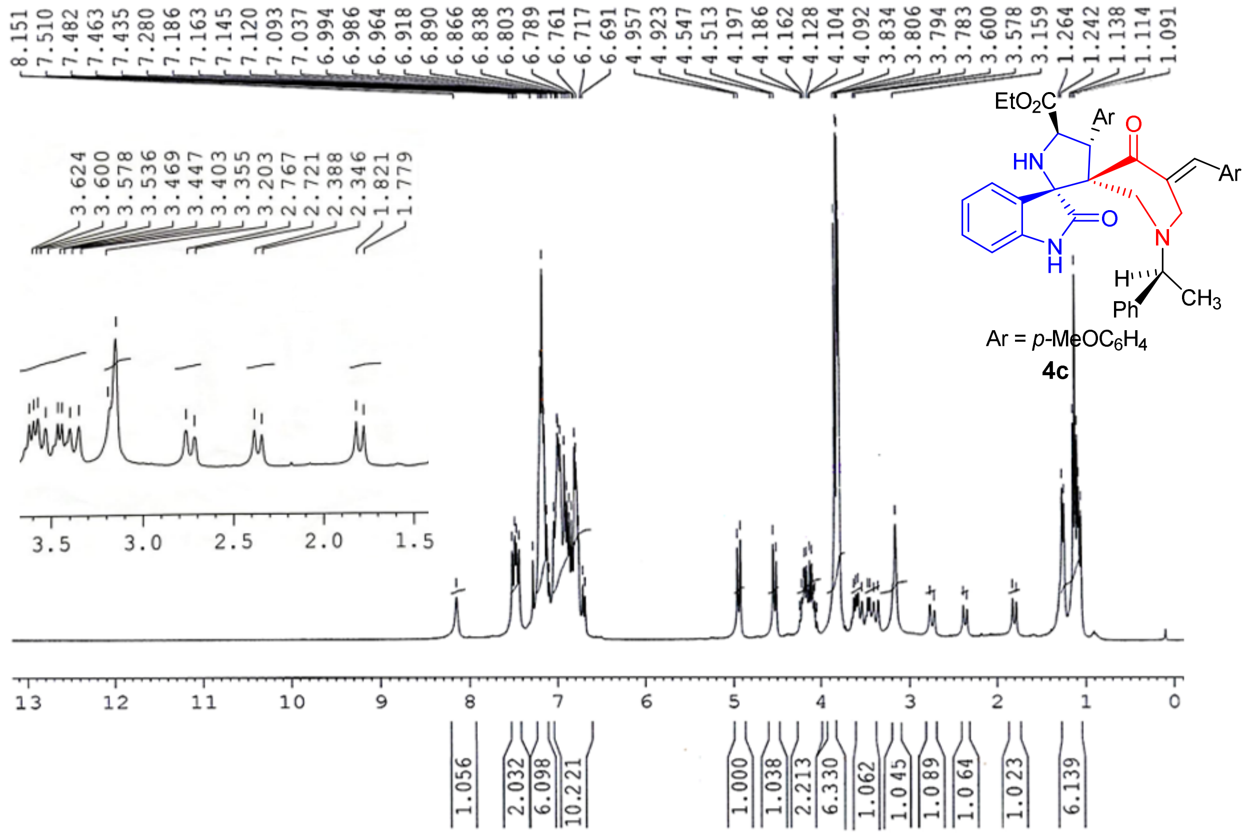

Figure S17. ^{1}H NMR spectrum of compound **4c** (300 MHz, $CDCl_3$)

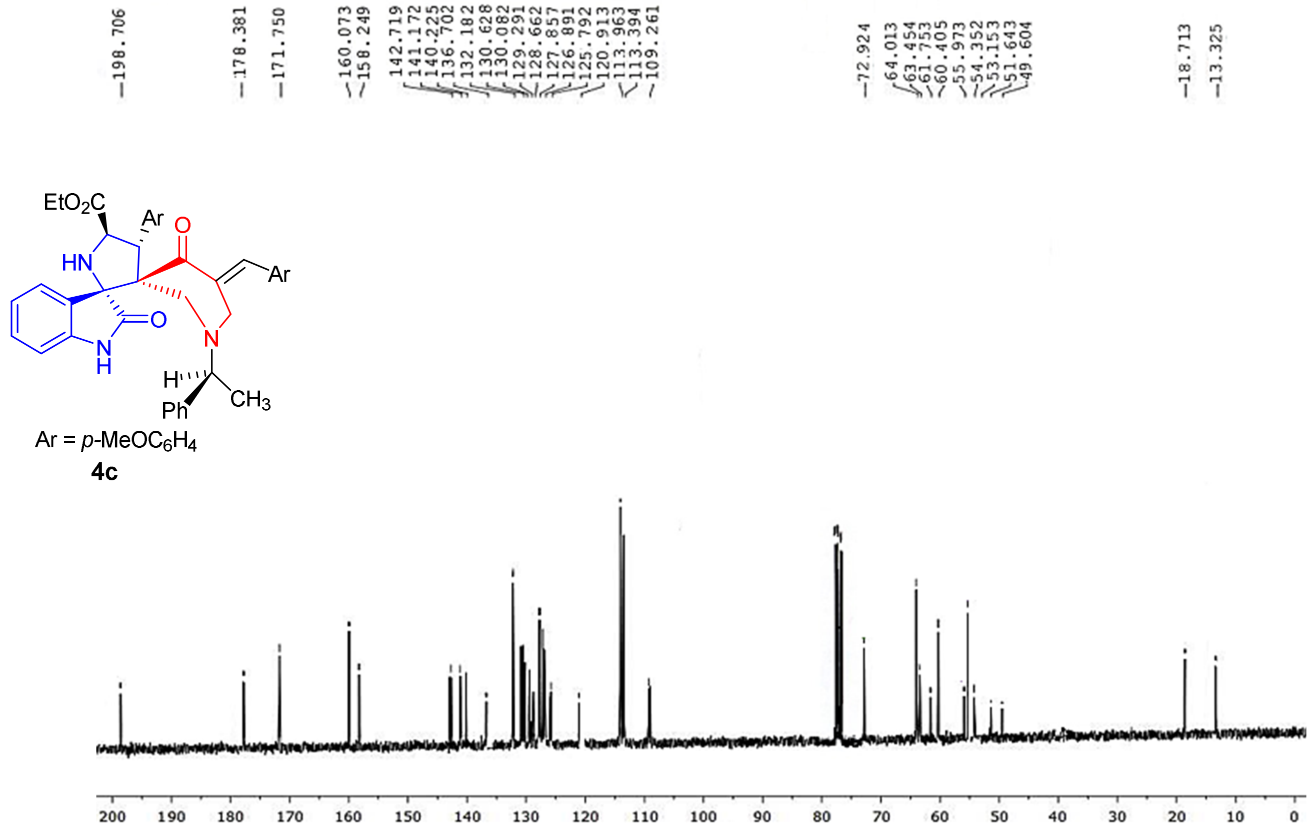

Figure S18. ^{13}C {^{1}H} NMR spectrum of compound **4c** (75 MHz, $CDCl_3$)

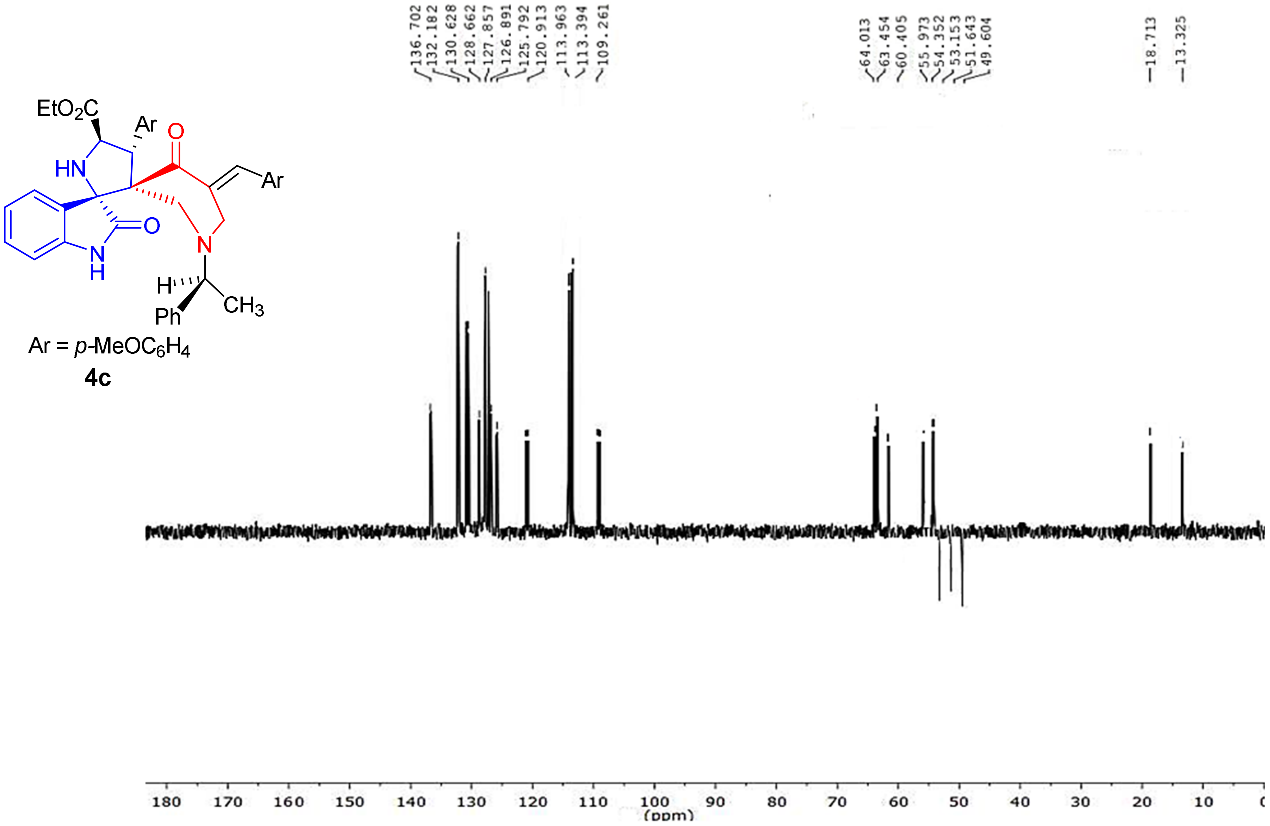

Figure S19. DEPT-135 spectrum of compound **4c** (75 MHz, $CDCl_3$)

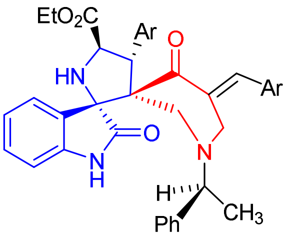

Ar = *p*-MeOC$_6$H$_4$

4c

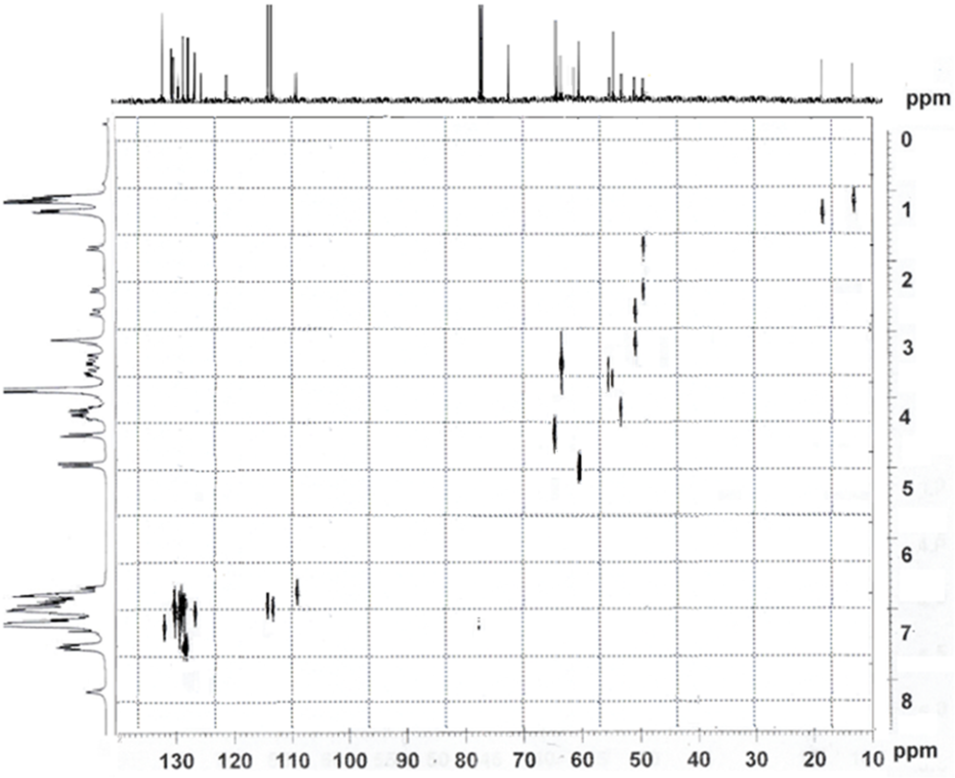

Figure S20. HMQC spectrum of compound **4c** (300 MHz, $CDCl_3$)

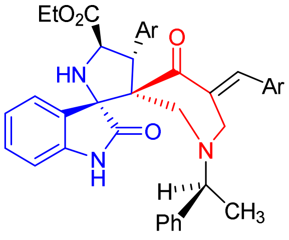

Ar = *p*-MeOC$_6$H$_4$
4c

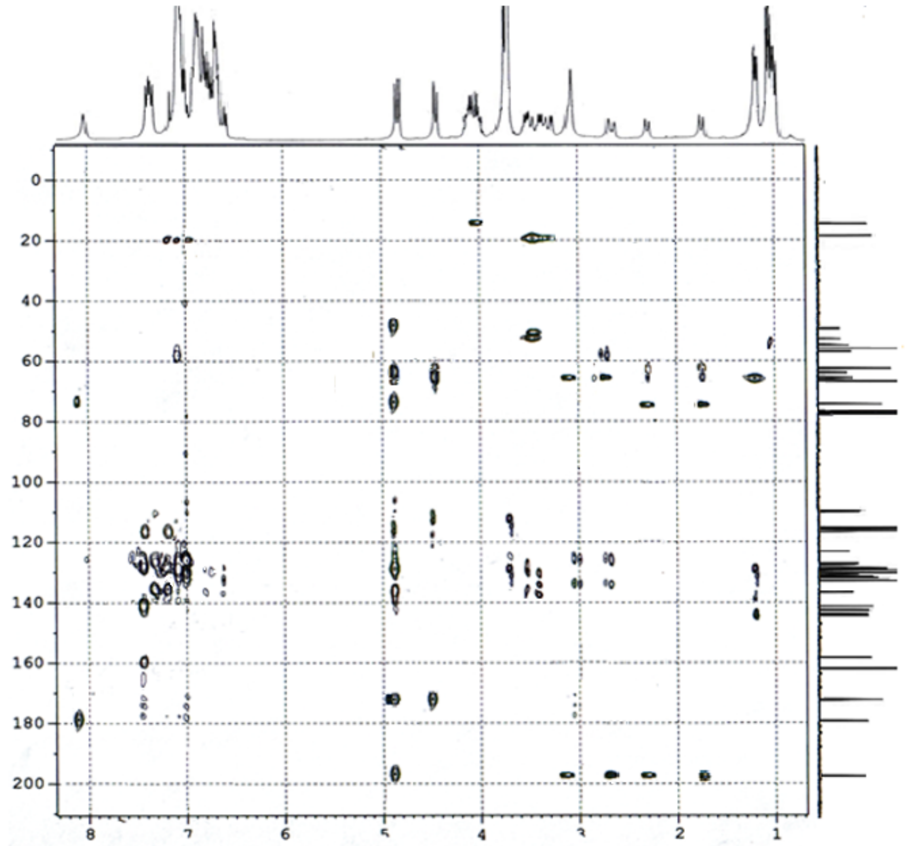

Figure S21. HMBC spectrum of compound **4c** (300 MHz, CDCl$_3$)

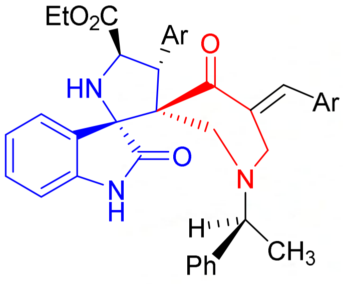

Ar = *p*-ClC$_6$H$_4$
4d

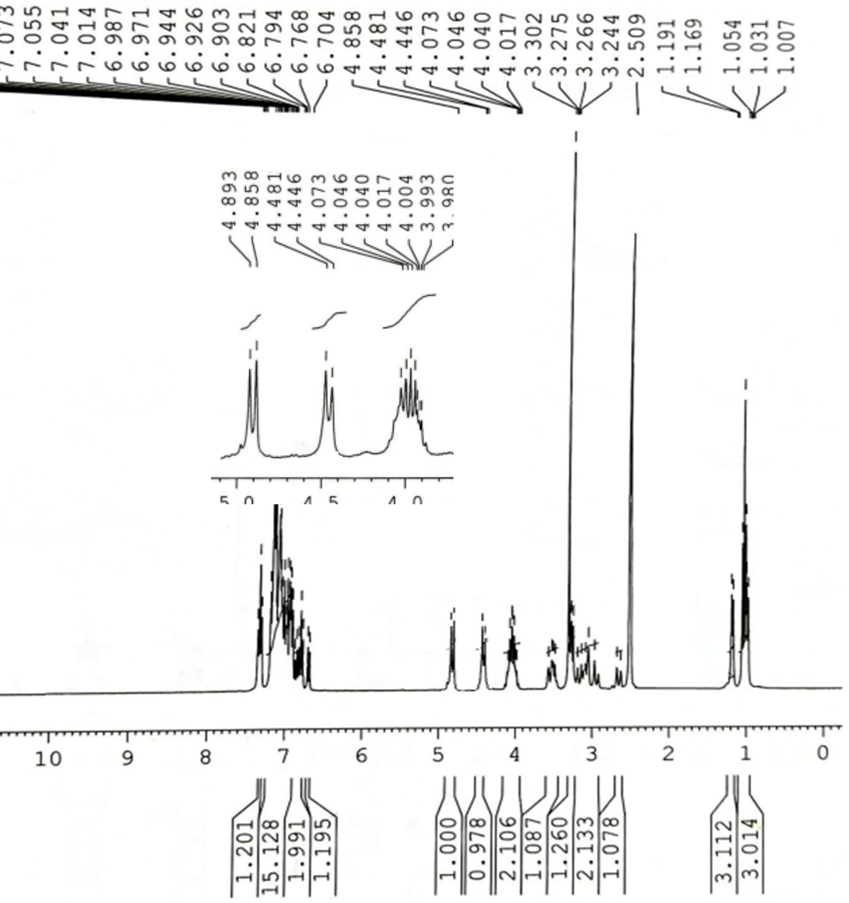

Figure S22. ^{1}H NMR spectrum of compound **4d** (300 MHz, DMSO-d_6)

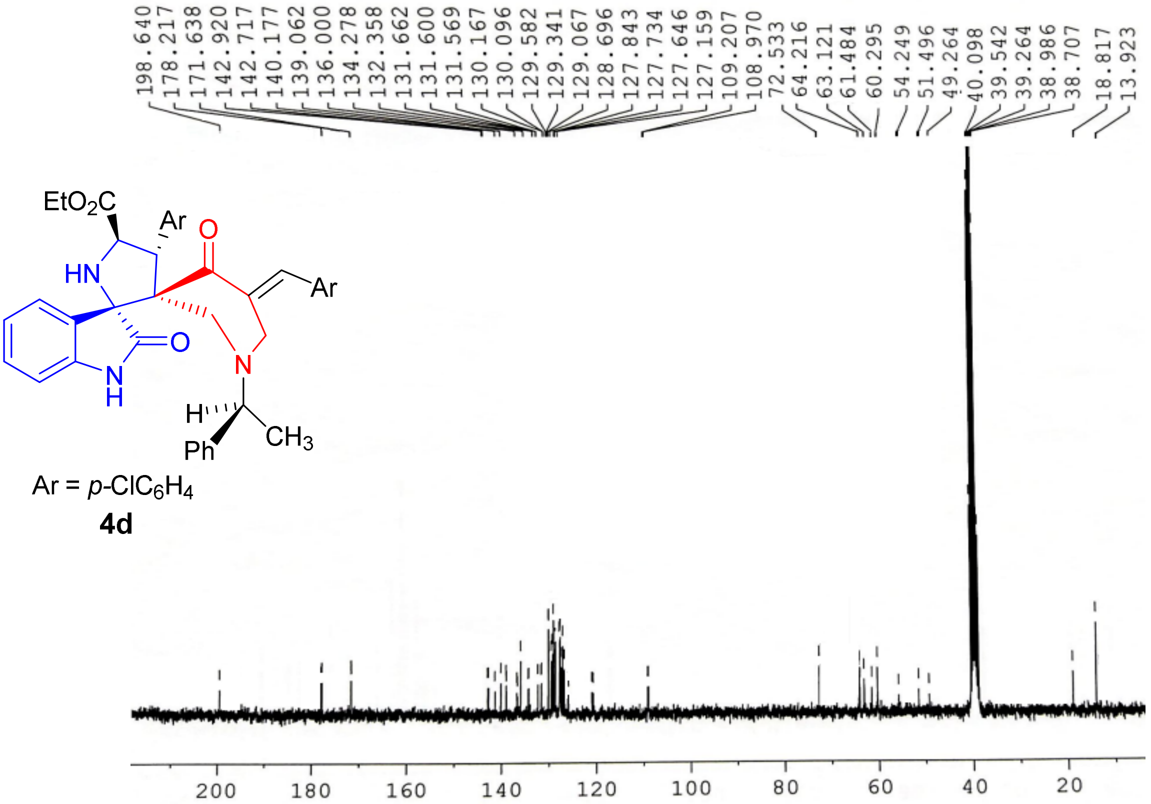

Figure S23. ^{13}C {^{1}H} NMR spectrum of compound **4d** (75 MHz, DMSO-d_6)

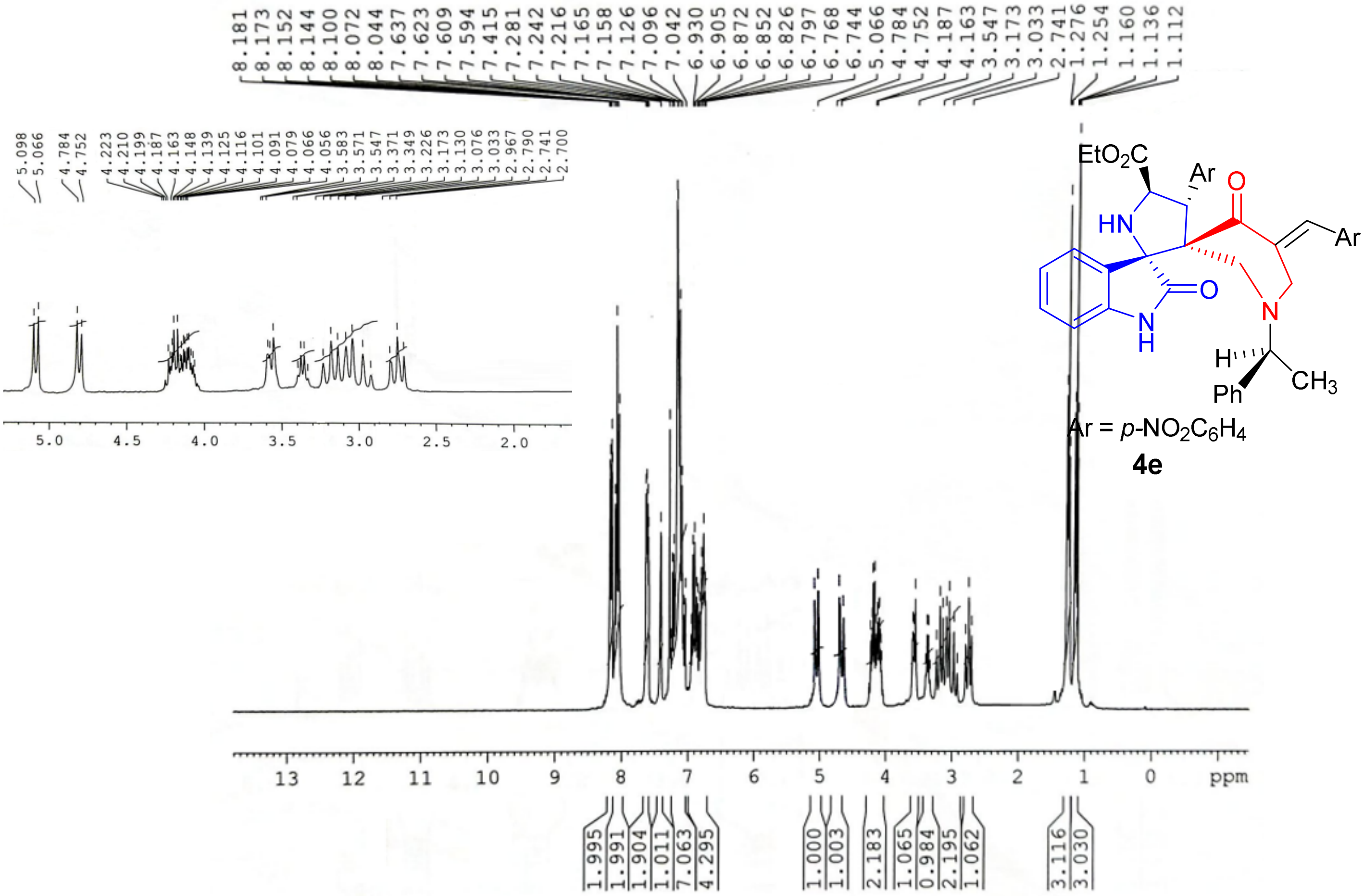

Figure S24. ^{1}H NMR spectrum of compound **4e** (300 MHz, $CDCl_3$)

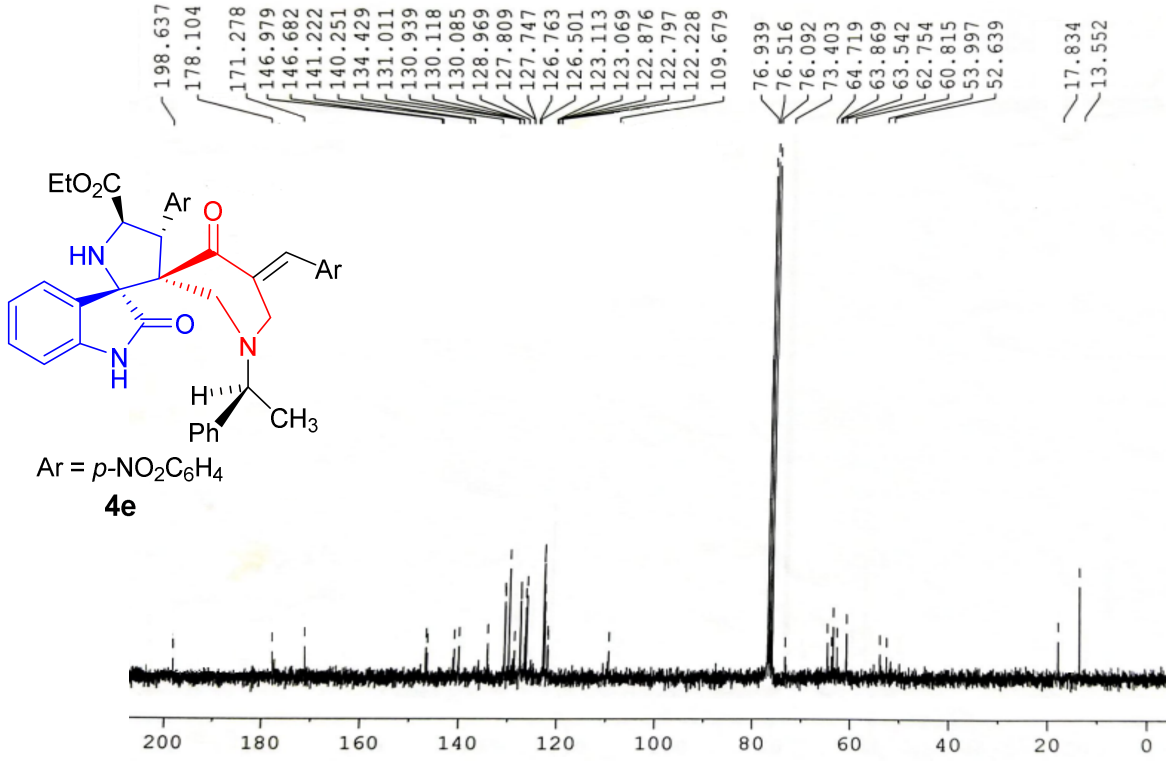

Figure S25. ^{1}H NMR spectrum of compound **4e** (300 MHz, $CDCl_3$)

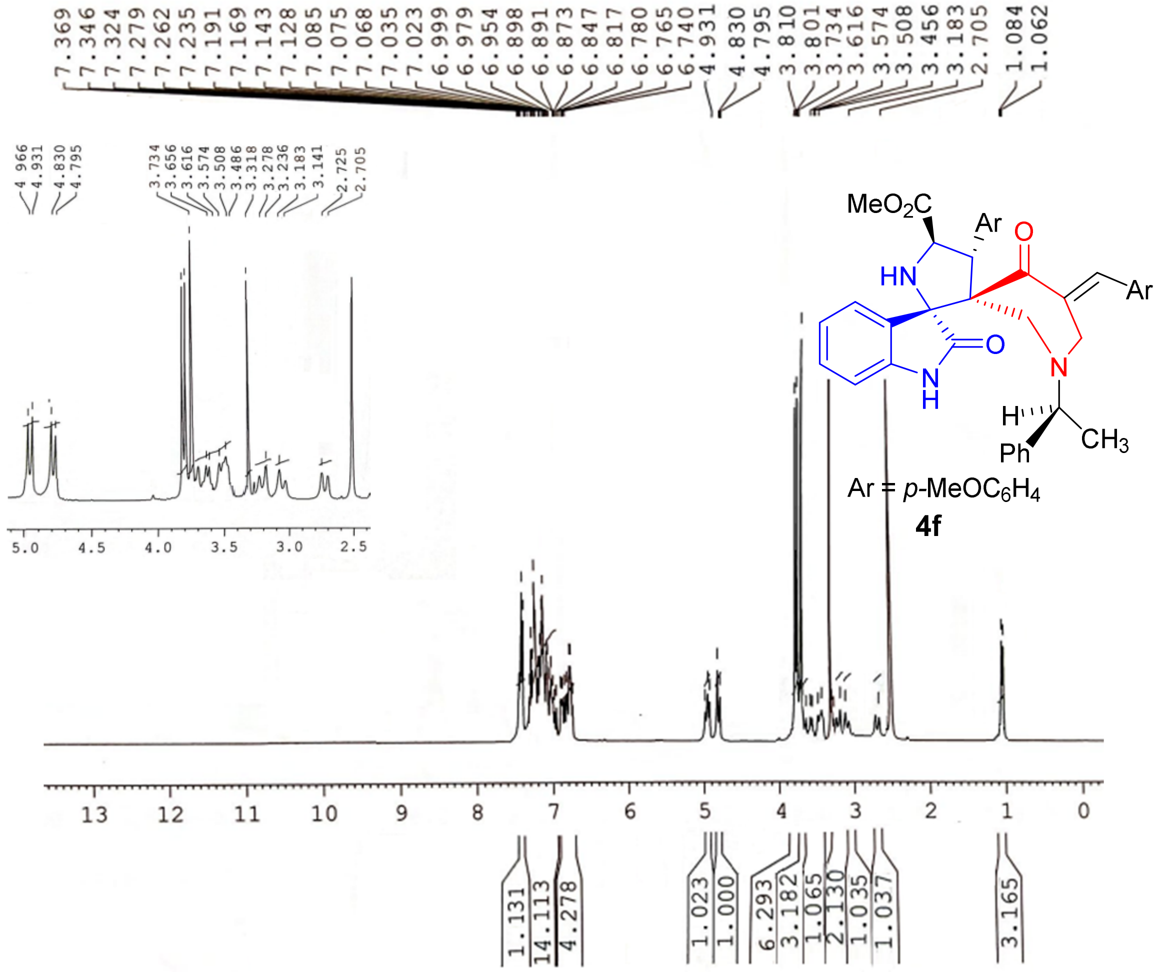

Figure S26. ^{1}H NMR spectrum of compound **4f** (300 MHz, DMSO-*d*6)

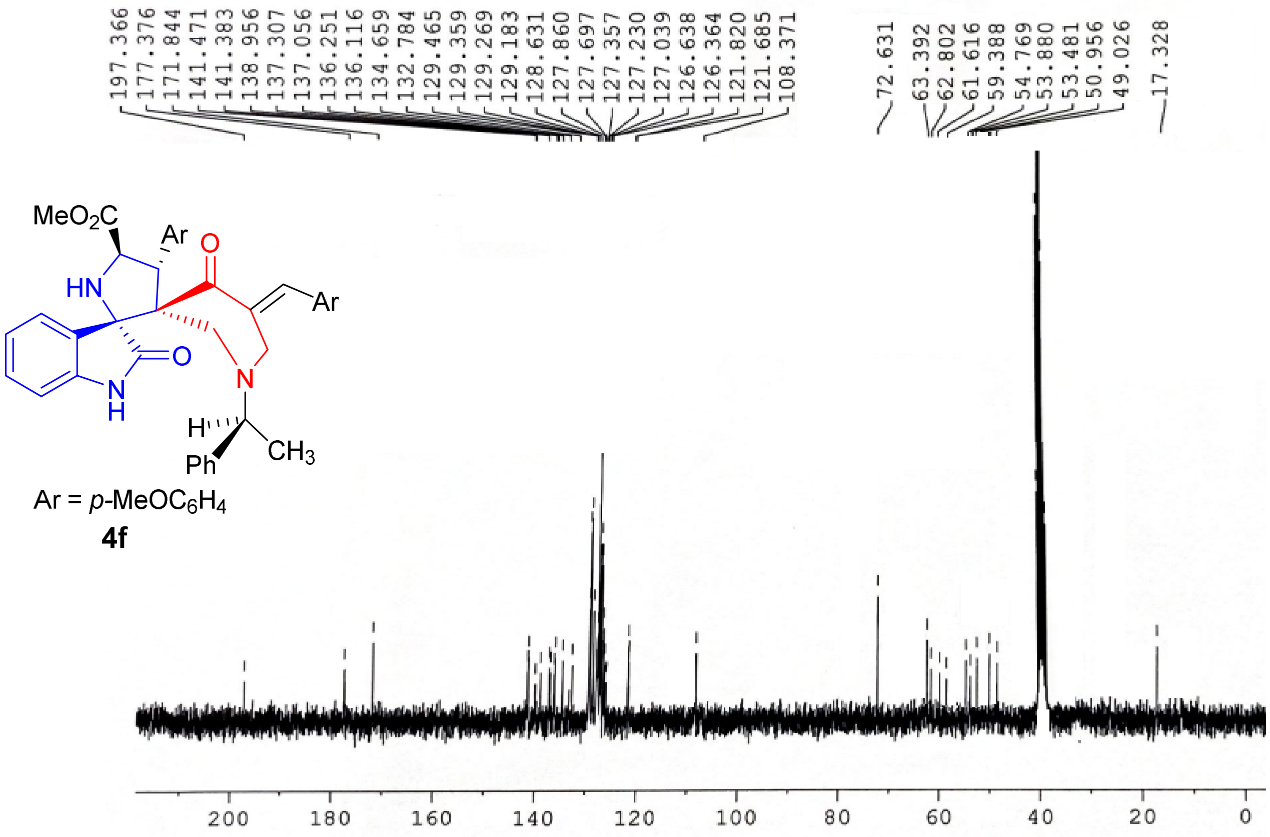

Figure S27. ^{13}C {^{1}H} NMR spectrum of compound **4f** (75 MHz, DMSO-*d*6)

Table S1. Selected bond lengths (Å) and angles for compound **5c**.

C1–N1	1.470(2)	C5–N2	1.359(2)
N1–C4	1.478(2)	N2–C6	1.393(3)
C1–C2	1.553(2)	C6–C11	1.404(3)
C2–C3	1.563(2)	C11–C4	1.519(2)
C3–C4	1.601(2)	C3–C12	1.532(2)
C4–C5	1.564(2)	C12–O2	1.226(2)
C5–O1	1.221(2)	C12–C13	1.494(2)
C14–N3	1.462(2)	N3–C15	1.456(2)
C13–C31	1.355(3)		
C1–N1–C4	103.87(14)	C14–N3–C15	113.38(14)
N1–C1–C2	105.65(14)	N3–C15–C3	107.79(14)
C1–C2–C3	103.41(13)	C15–C3–C12	106.88(14)
C3–C12–C13	116.31(15)	C12–C13–C31	116.85(16)
C13–C14–N3	115.37(14)	C13–C31–C32	129.99(17)

Table S2. Hydrogen Bonds for **5c.**

D–H···A	d(D-H)/Å	d(H-A)/Å	d(D-A)/Å	D-H-A/°
C1 –H1A ···O1	0.96(2)	2.40(2)	3.010(2)	121.0(16)
N2–H2···O11[1]	0.90(3)	1.96(3)	2.853(2)	169(3)
C56– H56A···O7	1.00(2)	2.40(2)	3.026(2)	120.3(17)
N5–H5···O5	0.87(3)	1.98(3)	2.846(2)	169(3)
